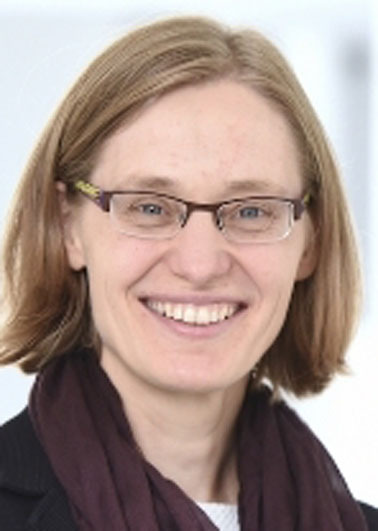# Droplet Microfluidics and Directed Evolution of Enzymes: An Intertwined Journey

**DOI:** 10.1002/anie.202016154

**Published:** 2021-07-16

**Authors:** Ariane Stucki, Jaicy Vallapurackal, Thomas R. Ward, Petra S. Dittrich

**Affiliations:** ^1^ Department of Biosystems Science and Engineering ETH Zurich Mattenstrasse 26 CH-4058 Basel Switzerland; ^2^ Department of Chemistry University of Basel Mattenstrasse 24a CH-4058 Basel Switzerland; ^3^ National Competence Center in Research (NCCR) Molecular Systems Engineering Basel Switzerland

**Keywords:** biocatalysis, directed evolution, droplet microfluidics, enzymes

## Abstract

Evolution is essential to the generation of complexity and ultimately life. It relies on the propagation of the properties, traits, and characteristics that allow an organism to survive in a challenging environment. It is evolution that shaped our world over about four billion years by slow and iterative adaptation. While natural evolution based on selection is slow and gradual, directed evolution allows the fast and streamlined optimization of a phenotype under selective conditions. The potential of directed evolution for the discovery and optimization of enzymes is mostly limited by the throughput of the tools and methods available for screening. Over the past twenty years, versatile tools based on droplet microfluidics have been developed to address the need for higher throughput. In this Review, we provide a chronological overview of the intertwined development of microfluidics droplet‐based compartmentalization methods and in vivo directed evolution of enzymes.

## Graphical abstract

From bulk emulsification to on‐chip droplet formation and sorting, droplet microfluidics offers numerous tools for high‐throughput screening. Directed evolution has greatly benefitted from these technological advances over the last twenty years. This review article gives a chronological overview of in vivo directed evolution of enzymes facilitated by droplet microfluidics.

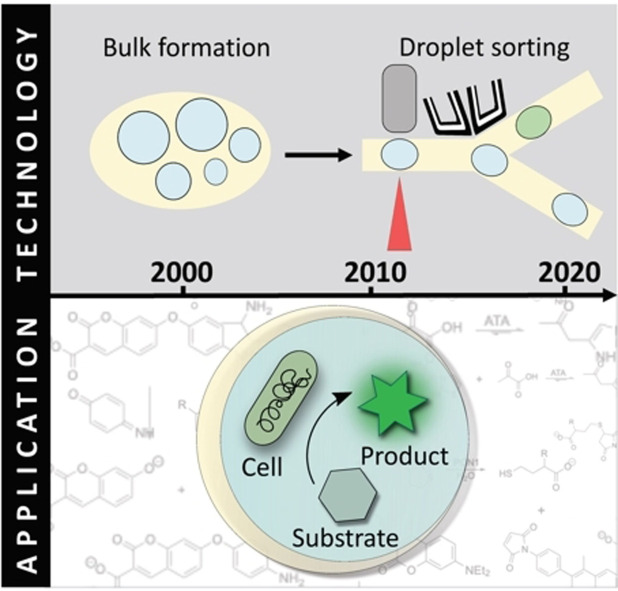

## 1 Introduction

Enzymes, nature's privileged catalysts, were optimized for a specific biological purpose and evolved over thousands of generations by natural selection. Lowering reaction barriers to selectively enable and accelerate certain reactions is a key characteristic of enzymes. But as the enzymes’ natural activities are often insufficient to meet the needs of mankind, artificial selection and screening have gained importance. Starting from the breeding of crops and domestication of animals for sustaining early populations, it matured to directed evolution in order to improve natural systems and introduce new‐to‐nature reactions for life sciences and other applications.

The 2018 Nobel Prize in Chemistry was awarded for efforts in the development of directed evolution to Frances H. Arnold for the directed evolution of enzymes, and George P. Smith and Sir Gregory P. Winter for the phage display of peptides and antibodies. Directed evolution makes it possible to alter and thus potentially improve biological activities by genetic means, an approach generally faster and with better control than natural selection. Accordingly, the study of these enzymes is of high importance to scientific advancement and holds great industrial potential, as it allows the evolution of alternative or new reaction pathways in a streamlined fashion. This approach thus provides environmentally friendly pathways to valorize enzymes as an alternative to the more traditional chemistry toolbox.^[1]^

Applying directed evolution consists of three steps: 1) to iteratively mutate (create genetic diversity), 2) screen (optimize for a desired property), and 3) choose (pick the best performing variant). If the protein of interest is well characterized, focused mutagenesis strategies can be implemented, followed by lower throughput screening.^[2]^ In a pioneering study, Arnold and co‐workers highlighted the potential of directed evolution using subtilisin E. By screening about 4000 colonies, they evolved a variant capable of hydrolyzing a peptide substrate with 256‐fold higher efficiency than wild‐type in 60 % dimethylformamide (DMF).^[3]^ Since then, numerous in vitro and in vivo studies based on focused libraries in microtiter plates (MTPs) have been reported.^[4]^ Selected examples include 1) the directed evolution of sortase A to improve its robustness and activity by focused loop engineering and head‐to‐tail backbone cyclization,^[5]^ 2) the directed evolution of enantiospecific enzymes,[^6^, ^7^, ^8^] 3) the directed evolution of P450 for various applications,[^9^, ^10^, ^11^, ^12^] and, more recently, 4) the directed evolution of a de novo designed retro‐aldolase,^[13]^ of a metalloenzyme for enantiospecific ester hydrolysis designed from short peptides,^[14]^ and of a metalloenzyme for olefin metathesis using an expanded nitrobindin variant.^[15]^ Lately, directed evolution finds also increased use in the biotechnological field: for example, the process and enzyme engineering approach applied to galactose oxidase for the biocatalytic transformation of 5‐hydroxymethylfurfural (HMF), a valuable building block in the synthesis of materials from renewable resources.^[16]^ Apart from MTP assays, another medium‐throughput approach is the use of agar plate based screening assays, which was illustrated with the directed evolution of transaminases as biocatalysts for chiral amine synthesis.^[17]^ Enclosing the enzymatic reaction within cells or immobilizing fluorescent products on the cell surface is yet another strategy to increase the throughput and was applied to several systems, such as the evolution of a P450 monooxygenase.^[18]^

**Figure 1:**
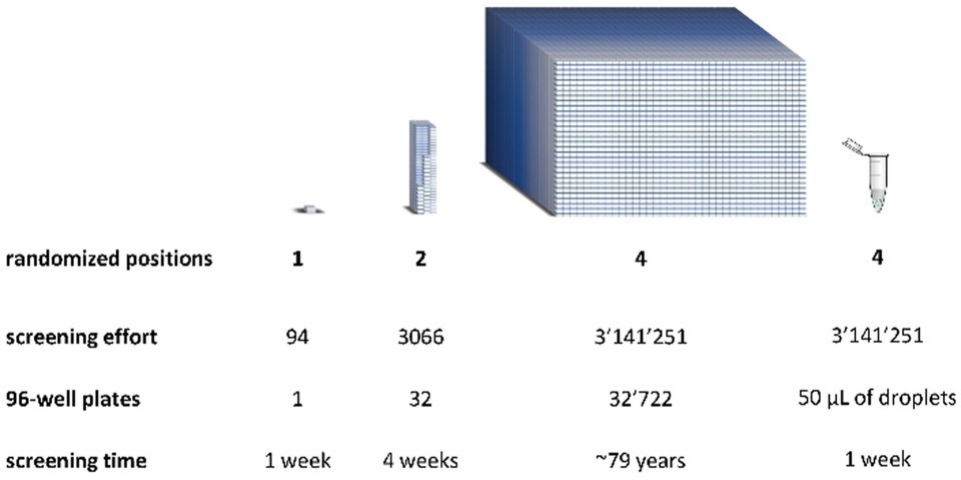
Schematic representation of the screening effort per mutated position using an NNK library. An NNK library at one position has 32 possible codons encoding for the twenty amino acids. This corresponds to a screening effort of 94 colonies to achieve a theoretical library coverage of 95 %. This effort increases exponentially if two or more positions are screened simultaneously. Screening four positions would require about 80 years, considering that eight 96‐well plates are screened per week. In comparison, screening the same library in double emulsions using microfluidic tools would require about one week of work.^[19]^

If the structure–activity relationships of the protein are poorly understood, more mutants may need to be screened to achieve a targeted phenotype. This is often achieved through a more thorough mutagenesis campaign of the protein and leads therefore to an exponential growth in the number of variants to be screened.^[19]^ Let us consider an example whereby four positions are simultaneously randomized. Using conventional screening assays based on MTPs may require over 80 years (roughly 20 PhDs!) of manual screening and appreciable amounts of material such as screening buffers (>100 L) and costly catalyst solutions (>1 L).^[20]^ In contrast, the same screening using double emulsions and fluorescence‐activated cell sorting (FACS) could be performed in roughly a week by a single operator with substantially lower amounts of material (Figure 1).

Additionally, to enable large screening efforts, optical readouts such as color, fluorescence, or luminescence are essential. In general, the industrially relevant target products lack readily detectable phenotypes. In such cases, substrate analogues with a fluorescent, luminescent, or colorimetric readout that correlates with the enzyme activity need to be implemented.

One of the main requirements in directed evolution is linking the activity of a target enzyme (i.e. the phenotype) to its genetic information (i.e. the genotype), which is essential for screening, selection and ultimately evolution campaigns.[^21^, ^22^] To address this challenge, different strategies, such as compartmentalizing the enzymatic reaction within/on cells or immobilizing fluorescent products on the cell surface, have been explored and are described in detail in other reviews.[^23^, ^24^] These strategies opened the way to high‐throughput analysis methods such as FACS. FACS devices have gained increasing interest since their initial development and the first instrument commercialization in the late 1960s–1970s.[^25^, ^26^] The development of microfluidic devices for fluorescence‐based particle‐ or cell‐sorting using negative dielectrophoresis (DEP) contributed to the early advancement of such technologies.^[27]^ Other fluorescence‐based methods such as fluorescent correlation spectroscopy (FSC) were developed around the same time for single‐molecule detection and analysis in solution and were further optimized with applications in evolutionary biology.^[28]^

Approaches where the fluorescent product remains in the cell or is immobilized on the cell are compatible with high‐throughput FACS but suffer from potential cross‐contamination and are incompatible with certain substrates. In vitro compartmentalization (IVC) in water‐in‐oil emulsions has emerged as an alternative to preserve the phenotype–genotype linkage.^[24]^ IVC has attracted a lot of interest and has been developed in parallel to the advancement of research on directed evolution over the past 20 years. Surfactant‐stabilized single (water‐in‐oil) or double (water‐in‐oil‐in‐water) emulsions (SEs and DEs, respectively) constitute optimal compartments for directed evolution thanks to their long‐term stability over a range of physicochemical factors including temperature, pH etc. Moreover, the formation of such compartments using microfluidic devices yields monodisperse droplets and allows for more controlled encapsulation of reactants.

**Figure 2:**
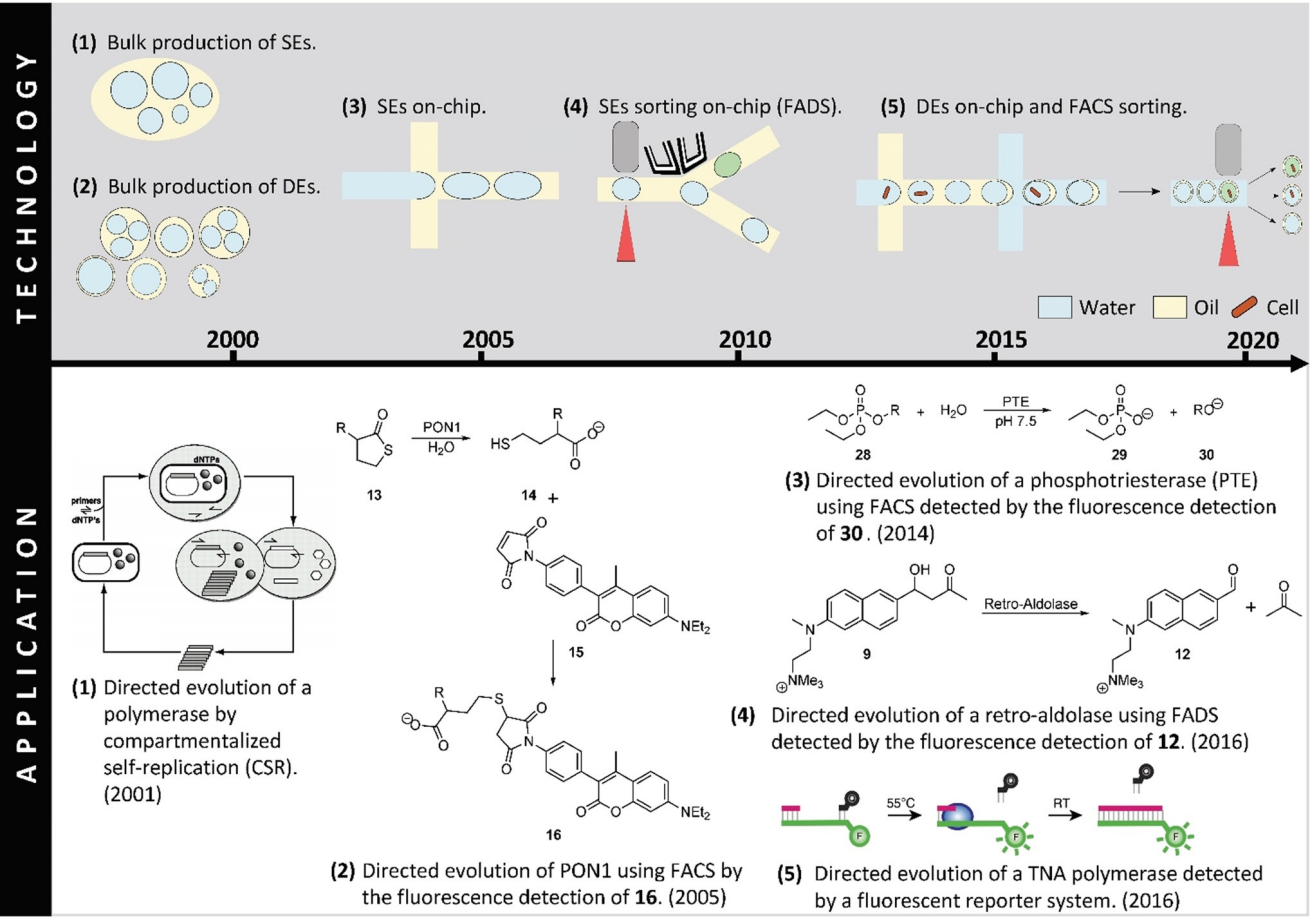
Milestones in the development of droplet microfluidics (top) and their applications to directed evolution (bottom) in the last twenty years. 1) Bulk production of single emulsions (SEs).^[29]^ Directed evolution of a Taq DNA polymerase based on compartmentalized self‐replication in SEs produced in bulk.^[30]^ 2) Bulk production of double emulsions (DEs).^[31]^ Directed evolution of *E. coli* surface‐displayed serum paraoxonase 1 (PON1) using DEs produced in bulk.^[32]^ 3) On‐chip production of SEs.[^33^, ^34^] Directed evolution of a phosphotriesterase through the encapsulation of *E. coli* expressing the enzyme on their surface in SEs produced on‐chip. The SEs consist of a gellable liquid and form gel beads following a gelation step. The beads can be analyzed and sorted by FACS.^[35]^ 4) On‐chip sorting of SEs.[^36^, ^37^] Directed evolution of a retro‐aldolase using SEs formed on‐chip and subsequent fluorescence‐assisted droplet sorting (FADS) on‐chip.^[38]^ 5) On‐chip formation of DEs followed by FACS sorting.[^39^, ^40^] Directed evolution of a manganese‐independent α‐L‐threofuranosyl nucleic acid (TNA) polymerase using DEs generated on‐chip and subsequent sorting by FACS.^[41]^ (Reprinted with permission (1) of the National Academy of Science USA. Copyright 2001; Reprinted (5) from ref. [41]).

Directed evolution studies have directly benefitted from the development of droplet microfluidics, allowing faster screening of larger libraries. In turn, the need for more specific and powerful tools for directed evolution has driven research in droplet microfluidics forward. In the last twenty years, engineering and biochemistry research groups have worked together to improve existing systems and develop new ones (Figure 2). In this Review, we provide a chronological overview of the intertwined development of microfluidics droplet‐based compartmentalization methods and in vivo directed evolution of enzymes.

## 2 Single Emulsions

Single‐emulsion droplets are aqueous compartments surrounded by an oil phase. The droplets can be stabilized using surfactants, i.e., amphiphilic molecules that arrange themselves at the water/oil interface. Methods of increasing complexity have been developed for the formation of such compartments, allowing improved control over the droplet size, the throughput, or the reagents’ encapsulation. Due to the external oil phase, water‐in‐oil (w/o) droplets are not compatible with commercially available FACS devices. To overcome this challenge, methods for on‐chip sorting have been implemented. Most recent devices have sorting throughputs of up to several kHz.^[42]^

### 2.1 Technology Advances I: Bulk Emulsification and Strategies for the Encapsulation and Immobilization of Reagents and Reaction Products

Different methods are used for the production of w/o compartments. Bulk emulsification allows fast and simple formation of droplets, but has limited encapsulation efficiency and yields polydisperse droplets. Bulk emulsification techniques, such as stirring and emulsifier‐based methods, were described before 1980.[^29^, ^43^] Later studies focused on the characterization of the physical properties of emulsions produced with custom‐made or commercially available homogenizers,[^44^, ^45^, ^46^] highlighting that droplets of sizes ranging from 0.1 to 100 μm in diameter can be produced.

**Figure 3:**
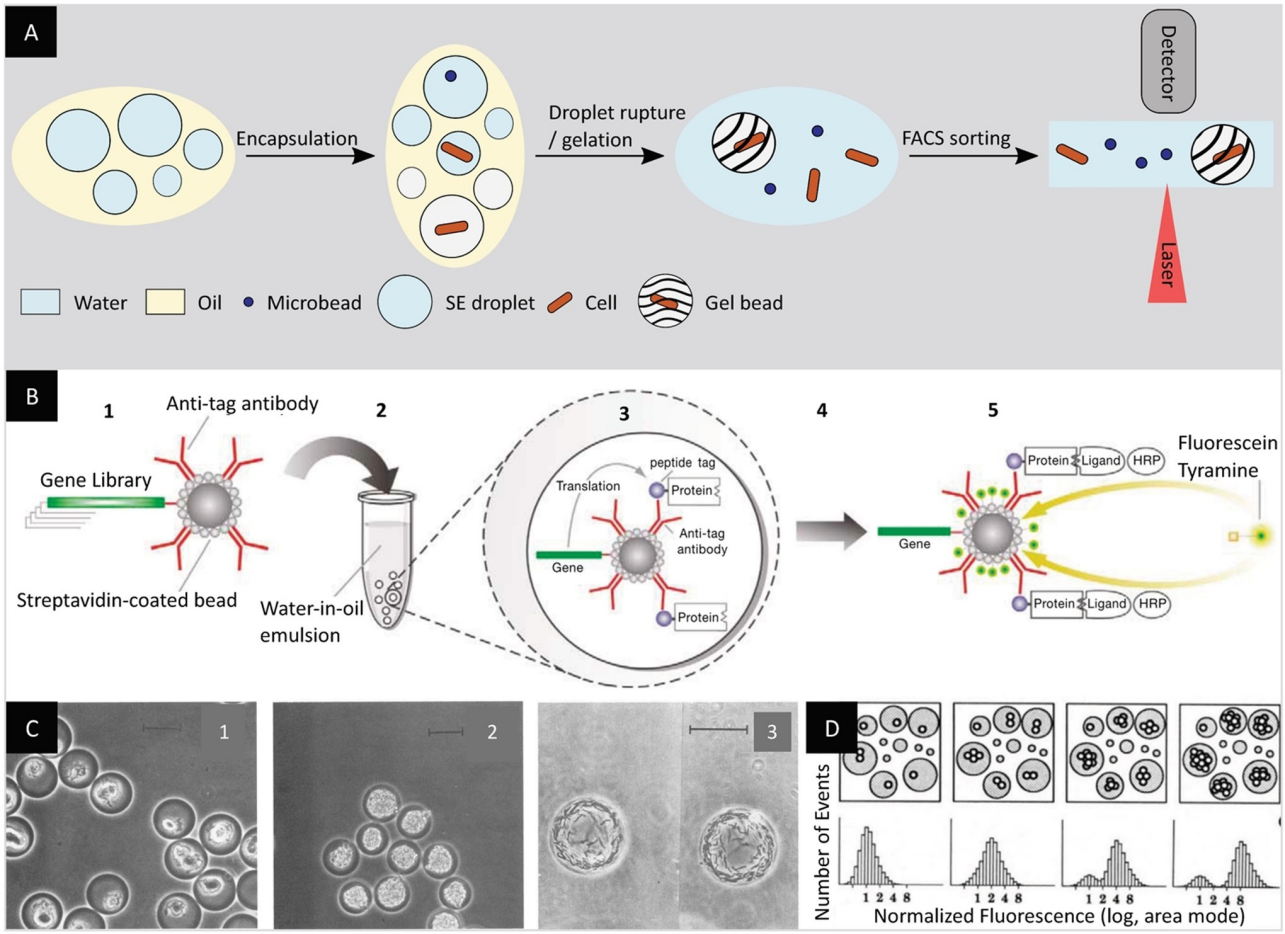
Bulk emulsification, encapsulation and sorting. A) Bulk emulsification permits the encapsulation in droplets of microbeads, cells, or genetic material together with substrates and reagents. Once the reaction of interest has been carried out, the product can be immobilized on the microbeads, cells, or in the droplet itself following a gelation process. The gel beads can be directly analyzed and sorted by FACS, while the beads and cells require the droplet to be ruptured first. B) Co‐encapsulation of an ivTT mixture with single DNA‐coated microbeads in droplets for the identification of proteins with high binding affinity towards a ligand via FACS sorting. (1) Streptavidin‐coated microbeads each displaying a variant of a gene library encoding the protein of interest (2) are co‐encapsulated with an ivTT mixture in w/o emulsions. (3) The translated proteins are immobilized by antibodies bound to the microbeads. (4) Droplet rupture permits the microbeads retrieval and (5) incubation with HRP, which binds to the proteins of interest. A second incubation step with hydrogen peroxide and fluorescein tyramide labels the beads fluorescently and permits FACS sorting for the identification of a protein with high affinity towards the ligand used in the screen.^[48]^ C) Micrograph of the encapsulation and growth of different microbial cells in gel microbeads: *E. coli* (1), *S. cerevisiae* (2), and *M. xanthus* (3). Scale bars: 20 μm.^[49]^ D) Fluorescence‐based biomass quantification of yeast cells trapped in gel microbeads.^[50]^ (Images reprinted with permission from (B) Wiley‐WCH Verlag GmbH & Co KGaA, (C) the American Society for Microbiology, (D) Springer Nature Limited.).

Whole cells or genetic material can be encapsulated in w/o droplets. The cell encapsulation follows the Poisson distribution and single‐cell compartmentalization can be achieved by adjusting the dilution of the cell‐containing solution.^[47]^ In the early 1990s, emulsions could be produced at high throughput but were incompatible with analytical tools with similar throughput. To circumvent this challenge, other droplet‐based strategies were developed to screen active variants with FACS devices. One of the first techniques to emerge consisted in co‐encapsulating an in vitro transcription and translation (ivTT) mixture with single microbeads, each displaying the gene encoding the protein of interest in w/o emulsions (Figure 3 B).^[48]^ In this study, antibodies bound to the streptavidin‐coated microbeads could immobilize the translated proteins. Upon translation, the emulsions were ruptured to retrieve the microbeads and, subsequently, incubated with horseradish peroxidase (HRP) which bound to the proteins of interest via a ligand. In a second step, the beads were incubated with hydrogen peroxide and fluorescein tyramide, leading to the fluorescent labeling of the bead. FACS sorting of the microbeads enabled the identification of a protein with high affinity towards the ligand used in the screen.

Another strategy enabling the use of FACS consists in generating droplets with a gellable liquid in which genes and either encoded enzymes or whole cells can be encapsulated. Through a cooling step, the droplets are converted into FACS‐compatible gel beads, immobilizing and compartmentalizing the genetic material (Figure 3 C).^[49]^ The relative permeability of gel beads favors the constant intake of growth medium or the addition of certain substrates at a later time point, while retaining the cell microcolonies. Using this technique, Weaver et al. encapsulated mammalian, bacterial, and fungal single cells in agarose beads with diameters of 20 to 90 μm (Figure 3 D).^[50]^ After an incubation step in the growth medium and a staining step with fluorescent markers for biomass, the cell colonies were analyzed by FACS. In a related study, Sahar et al. analyzed the properties of the encapsulated bacterial colonies.^[51]^ Among others, they characterized the intracellular esterase activity of a *P. aeruginosa* cell population. This was achieved through the addition of a fluorogenic substrate, 6‐carboxyfluorescein‐diacetate, to the gel beads followed by an incubation step. They additionally described the activity of the secreted enzyme elastase by encapsulating its fluorescently labeled substrate casein during droplet formation and determined the decrease in fluorescence caused by the leakage of the product out of the bead.

#### 2.1.1 Applications I: In Vitro

**Figure 4:**
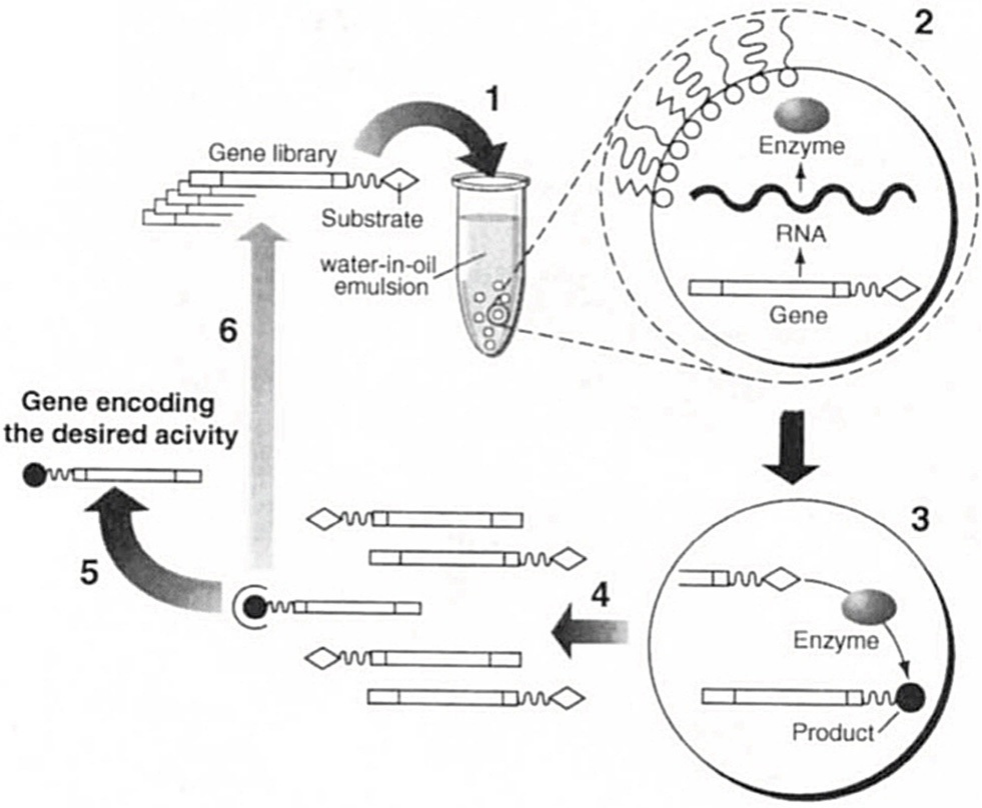
Schematic representation of an IVC selection strategy for DNA/RNA. (1) One single gene linked to a substrate is compartmentalized within a w/o emulsion. (2) ivTT yields a functional protein/RNA and (3) an enzymatic reaction converts a substrate into a product which remains linked to the gene. (4) After the emulsions are ruptured, (5) the genes linked to the product are selectively enriched and (6) either characterized and/or encapsulated for another round of evolution.^[52]^ (Reprinted with permission from Springer Nature Limited.).

The first study using in vitro compartmentalization for applications in molecular evolution resulted from a collaboration between the Griffiths and Tawfik groups.^[52]^ In this study, in vitro compartmentalization (IVC) of a single gene encoding either a DNA‐methyltransferase HaeIII or a dihydrofolate reductase (DHFR) followed by ivTT led to the enrichment of an enzyme for DNA methylation (Figure 4). M.HaeIII genes encoding HaeIII, and folA genes encoding DHFR, both containing a site designed for methylation/restriction by M.HaeIII, were encapsulated and tested for methylation efficiency. If M.HaeIII was present, the gene was methylated and was thus not digested in the subsequent digestion step with the endonuclease HaeIII. On the other hand, if DHFR was present, the gene was not methylated and was therefore digested by the HaeIII endonuclease.

Methylated HaeIII sites resistant to digestion were amplified using PCR and analyzed on an agarose gel. Model enrichment of a library starting with 0.1 % M.HaeIII led to a 500‐fold enrichment in a single cycle. The same approach was used in a follow‐up study to improve the sequence specificity. A more active species was selected from a random mutagenesis library at three positions with ≈3.3×10^7^ variants. Remarkably, over only two rounds of screening, 11 variants with up to ≈19‐fold improvement were identified. All identified hits bore two mutations, whereas the third position proved to be crucial for the methyltransferase activity and did not tolerate any other mutation.^[53]^

**Scheme 1:**
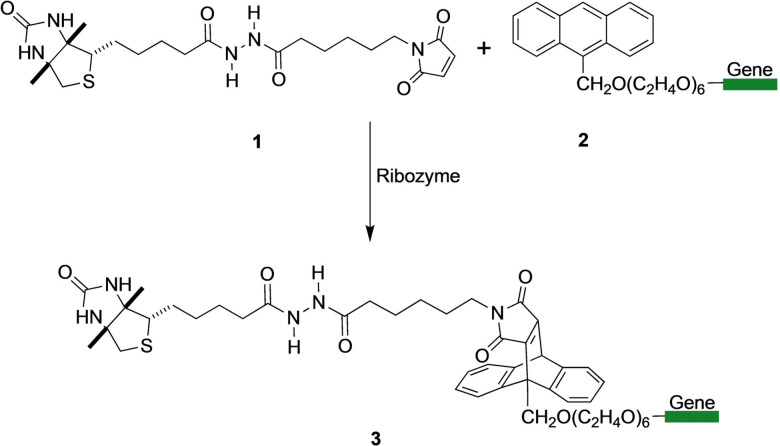
The intermolecular Diels–Alder reaction between biotin‐maleimide (**1**) and AHEG (**2**). The gene to be evolved is covalently attached to **2** and **3**, but only active catalysts give products **3**. The Diels–Alder products **3** are subsequently captured by streptavidin‐coated magnetic beads, allowing their downstream PCR enrichment.^[56]^

With the aim of bringing the technology to the next level, single emulsions were used to evolve ribozymes for a bimolecular Diels–Alder reaction. In a larger evolution campaign consisting of four rounds of IVC, ribozymes catalyzing the intermolecular Diels–Alder reaction between 9‐anthracenylmethyl hexaethylene glycol (AHEG, **1**) and biotin‐maleimide (**2**) with multiple turnovers were evolved (Scheme 1). After four rounds of evolution, variants with a catalytic efficiency *k*_cat_/(*K*_m1_ K_m2_)=5.3×10^5^ M^−2^ s^−1^ were identified. These artificial enzymes display efficiencies that are comparable to catalytic Diels–Alderase antibodies.^[54]^ Using a custom‐built homogenizer, Paegel and Joyce evolved RNA enzymes with ligase activity, selecting enzymes that could resist inhibition by neomycin. A library of 10^11^ variants was evolved over five rounds to obtain mutants with better tolerance to neomycin and generally higher *K*_m_ values.^[55]^

#### 2.1.2 Applications II: In Vivo

**Figure 5:**
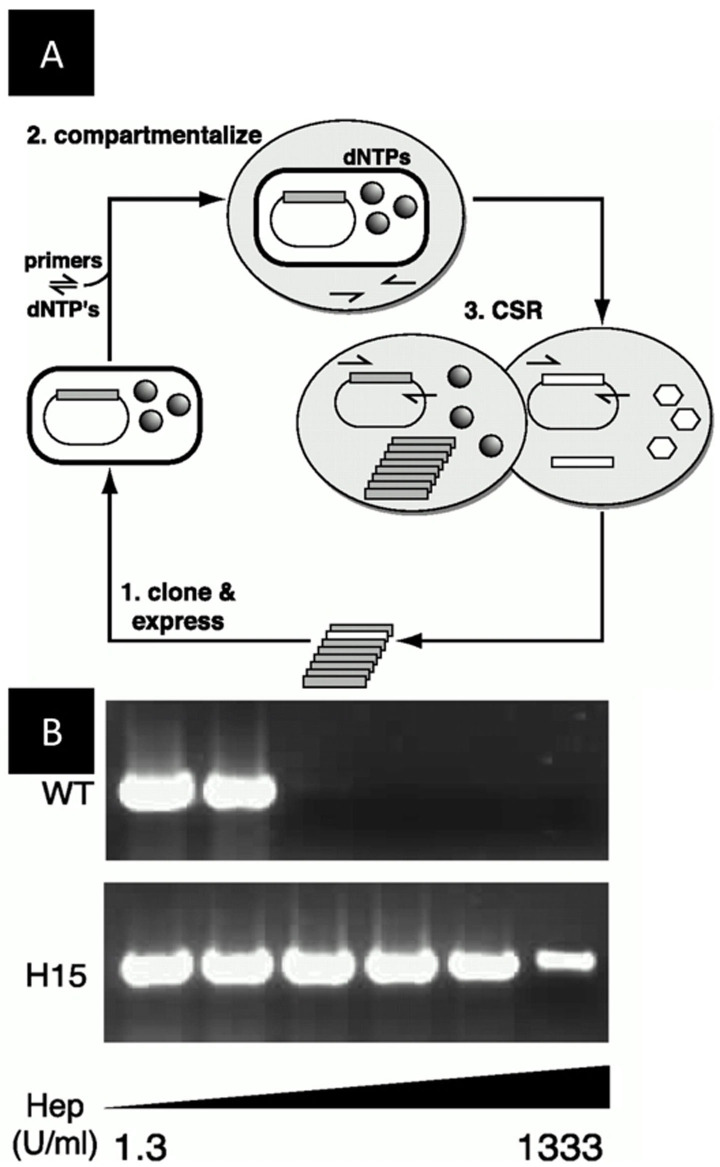
First in vivo directed evolution study in single emulsions based on CSR. A) Compartmentalized self‐replication (CSR): (1) Genes encoding a polymerase are cloned and expressed in *E. coli* and encapsulated in w/o droplets together with primers and dNTPs (2). Poorly active variants (hexagons) cannot replicate efficiently, whereas functional and active variants of the polymerase enzyme (spheres) result in self‐replication (3) and can be extracted and analyzed or recloned for another cycle of CSR. B) PCR activity of the wild type and evolved mutant (H15) in the presence of heparin.^[30]^ (Reprinted with permission from National Academy of Science USA. Copyright 2001.).

Meanwhile, the first in vivo applications using single emulsions were reported. The screening approach used for the first studies was based on compartmentalized self‐replication (CSR) (Figure 5 A). The directed evolution of Taq DNA polymerase was carried out in polydisperse emulsions generated by stirring. With this approach, Ghadessy et al. identified a Taq DNA polymerase variant with elevenfold increased thermostability and a variant with over 130‐fold increased resistance to the inhibitor heparin (Figure 5 B).^[30]^ Other similar approaches of CSR in single emulsions involved the directed evolution of the same Taq polymerase for broader substrate scope and faster‐cycling mutants (35–90‐fold higher affinity for the primer, twofold increase in extension rate).^[57]^ To expand the technology to non‐polymerase type enzymes, cooperative CSR was applied to evolve a nucleoside diphosphate kinase (NDK). NDK converted dNDPs to dNTPs which, in turn, could be used by a polymerase to replicate the genetic material. In this manner, only genes encoding active NDK were replicated, thus affording a straightforward approach to evolve simple cascade reactions.^[30]^

**Figure 6:**
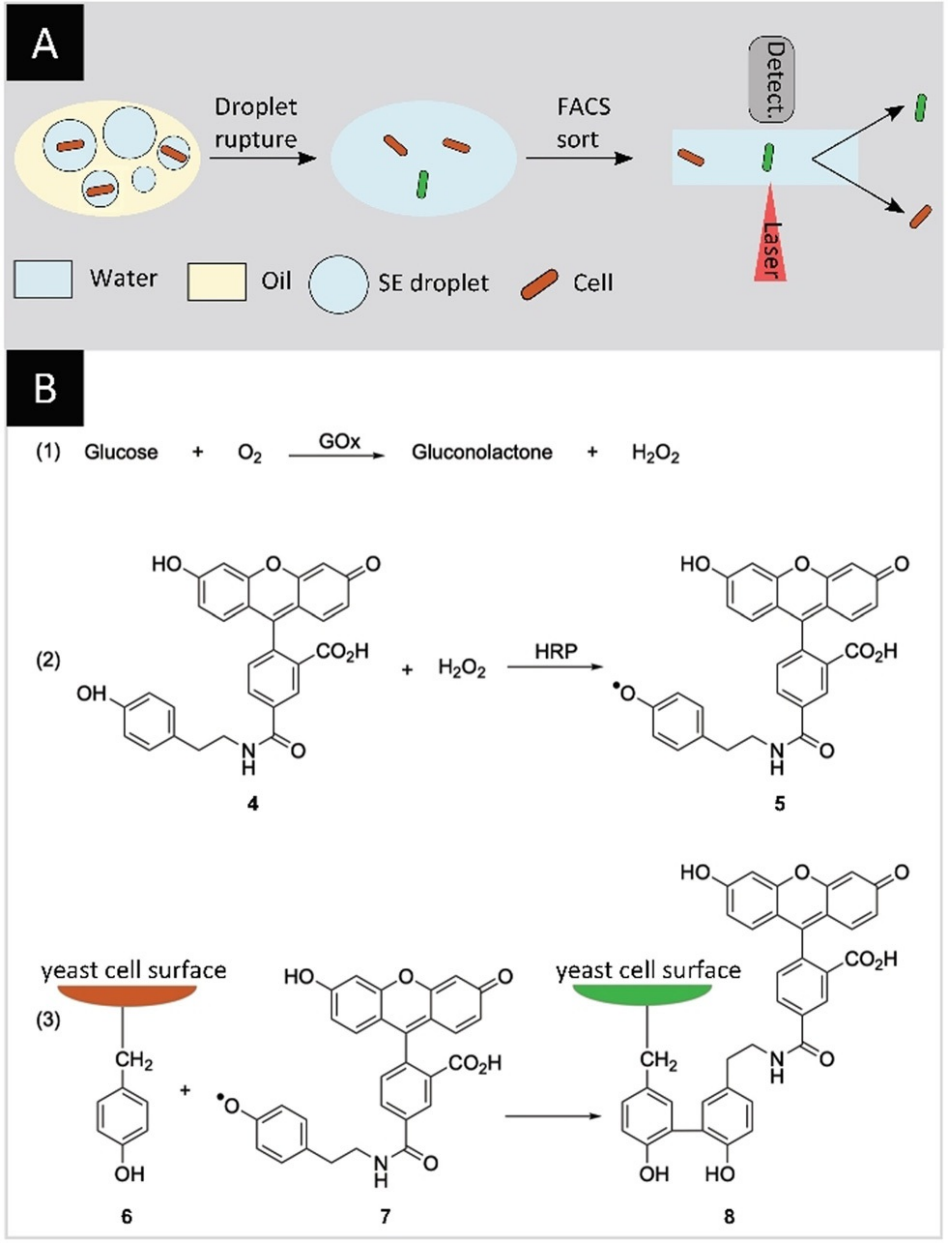
Screening of yeast surface‐displayed GOx. A) Yeast cells with surface‐displayed GOx and HRP are encapsulated in w/o emulsions. The catalytic reaction leads to stained yeast cells which, after rupturing the emulsions, are amenable to FACS sorting. B) GOx converts glucose into gluconolactone (1) and the byproduct H_2_O_2_ is reduced by the HRP to produce a fluorescein tyramide radical **5** (2). The radical then reacts with a tyrosine residue **6** on the surface of the yeast cell, leading to a stained yeast cell **8** (3).^[58]^

The systems described above rely mostly on self‐replication. Retaining a fluorescent signal on the encapsulated species itself is an essential feature to allow for FACS sorting. This was illustrated with yeast cells encapsulated in droplets. A library of yeast cells with surface‐displayed glucose oxidase (GOx) and horse radish peroxidase (HRP) was encapsulated and screened for the conversion of glucose to gluconolactone (Figure 6). The hydrogen peroxide byproduct of this reaction was reduced by HRP, leading to the generation of a fluorescein tyramide radical which, in turn, reacted with a tyrosine residue on the surface of the yeast cell. In this manner, the yeast cells retained the fluorescent information and could be sorted after rupturing the emulsions. From a library containing 10^5^ variants, resulting from error‐prone polymerase chain reactions (epPCR), a variant with five mutations and a 2.7‐fold improvement in *k*_cat_ was identified.^[58]^ Similarly, GOx was evolved for different conditions, resulting in twofold improved thermal stability compared to wild type (*t*_1/2_ ≈20 min at 60 °C) as well as a fourfold and 5.8‐fold improvement in *k*_cat_ at pH 5.5 and pH 7.4 respectively.^[59]^

Recently, GOx was coupled to the yeast‐enhanced green fluorescent protein (yGFP) to afford a chimera allowing the simultaneous detection of the protein expression level and the activity of the same enzyme. This system led to a 2.5‐fold enrichment of expressed, active variants and a 2.3‐fold increase in *V*_max_ in just one round of screening.^[60]^

#### 2.1.3 Applications III: Encapsulated Microbeads

A prominent example involving microbeads consists of the directed evolution of an extremely efficient phosphotriesterase (PTE) using streptavidin‐coated microbeads.^[35]^ In this study, Griffiths and Tawfik used polystyrene microbeads displaying single genes anchored via a biotin‐streptavidin linkage. Within the w/o emulsions, multiple copies of PTE were produced by ivTT and anchored to the bead using an antibody. The emulsions were then ruptured and the beads were re‐encapsulated to add a soluble biotin‐tagged substrate. The catalysis was performed inside the emulsions and the biotin‐tagged product was retained on the bead. Subsequent rupture of the emulsions and labeling with a fluorescent anti‐product antibody facilitated the sorting of active species. Relying on this approach, the authors identified a variant with a *k*_cat_ of 10^5^ s^−1^ after six rounds of directed evolution from a library of 3.4×10^7^ variants. This corresponds to a 63‐fold improvement over the wild type enzyme. This work paved the way for similar approaches such as the enrichment of an oxygen‐tolerant [Fe‐Fe] hydrogenase I from *C. pasteurianum* (CpI) for the reduction of the fluorogenic compound C_12_‐resazurin^[61]^ and the mock enrichment of a wild type HRP immobilized on microbeads.^[62]^ Notable directed evolution efforts include the directed evolution of 1) a *trans*‐acting Bartel class I ligase with up to 90‐fold rate enhancement^[63]^ and 2) a sortase A from *Staphylococcus aureus* with a 114‐fold enhancement in *k*_cat_/*K*_m_.^[64]^

Recently, Panke and co‐workers used alginate beads for the directed evolution of the broad‐spectrum amino acid racemase from *Pichia pastoris* (*Pp*AAR) for the racemization of d‐ornithine, an interesting target for industrial applications. Starting from a library with 1.2×10^7^ variants, they observed an up to 2.7‐fold *k*_cat_/*K*_M_ improvement over wild type after three rounds of directed evolution.^[65]^

### 2.2 Technology Advances II: Microfluidics‐Based Droplet Formation and Reduction of Cross Talk between Droplets

**Figure 7:**
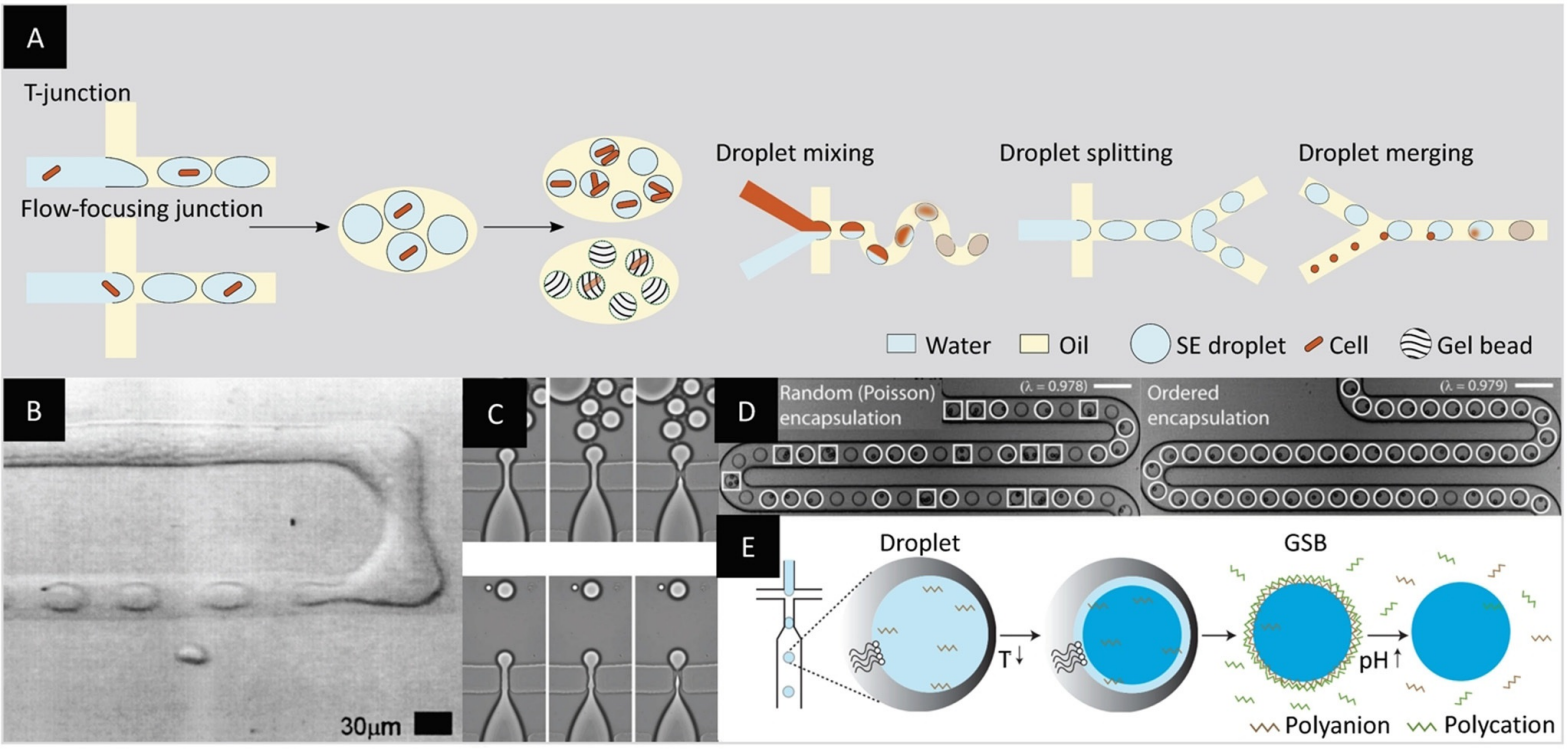
Microfluidics‐based formation of monodispersed w/o emulsion droplets. A) The use of microfluidic chips with channels forming either a T‐junction or a flow‐focusing junction allows the formation of highly monodisperse droplets at high throughput. Specific channel geometries can be used to improve droplet mixing, splitting, or merging. B) Micrograph of the first T‐junction design for droplet production at 20–80 Hz.^[33]^ C) Micrograph of the first PDMS chip with flow‐focusing junction for the formation of droplets as small as 10 μm in diameter.^[34]^ D) Micrograph of the comparison between random bead encapsulation in droplets with improved bead encapsulation using cell alignment resulting from hydrodynamic interactions. Droplets containing two particles or more are highlighted by a square while droplets encapsulating single particles are highlighted by a circle. Scale bars: 100 μm.^[70]^ E) Microfluidic production of droplets with a gellable liquid for the formation of gel beads with a semipermeable polyelectrolyte shell (gel‐shell beads, GSBs). The shell can be ruptured under basic conditions.^[73]^ (Images reprinted with permission from (B) American Physical Society, (C) AIP Publishing LLC, (D) The Royal Society of Chemistry, (E) Springer Nature Limited.).

The need for higher droplet monodispersity and better control over the formation process led to the development of the first microfluidic chips for droplet production.[^66^, ^67^] Compared to bulk emulsion methods, microfluidics‐based methods require the fabrication of the device and its operation but allow for high‐throughput encapsulation in monodispersed compartments. The first study by Thorsen et al. introduced a device with a T‐shaped junction to generate emulsions at a frequency of 20–80 Hz (Figure 7 B).^[33]^ Similarly, Anna et al. displayed the controlled formation of droplets in a flow‐focusing channel geometry for the production of emulsions with droplet diameters as small as 10 μm (Figure 7 C).^[34]^ Similar channel geometries were used later to analyze reagent mixing inside droplets, spontaneous merging of droplets of different sizes, reagent addition to droplets, and droplet splitting.[^68^, ^69^]

For directed evolution, each droplet ideally contains one cell. However, cell encapsulation using microfluidic devices follows the Poisson distribution, resulting in a majority of empty droplets at low cell concentrations, thus lowering the effective throughput. Yet, unlike bulk methods, several studies have highlighted the possibility of overcoming Poisson's distribution limitations on a microfluidic device. Using particular channel geometries and hydrodynamic effects at high flow rates to order cells, various groups succeeded in yielding up to 80 % single‐cell‐containing droplets (Figure 7 D).[^70^, ^71^]

Another critical aspect of the compatibility of droplet microfluidics with directed evolution resides in the ability of the droplets to retain the substrate and product of the enzymatic reaction of interest. In their study, Courtois et al. investigated the leakage of fluorescein‐based substrates from droplets into the oil and succeeded in improving the retention to more than 18 hours by addition of bovine serum albumin (BSA). The authors illustrated the versatility of their system by characterizing the enzymatic activity of alkaline phosphatase expressed by *E. coli* cells and distinguishing empty droplets from cell‐containing droplets.^[72]^ The retention of substrate and product in w/o emulsions can also be addressed with the use of gel beads as described earlier, which can be created as well by droplet microfluidics (Figure 7 E).^[73]^

#### 2.2.1 Applications I: In Vitro and In Vivo

As early as 2013, Scanlon et al. presented the application of hydrogel emulsions produced on chip for the discovery of natural product based antibiotics. A recombinant antibiotic‐producing microbe (*Saccharomyces cerevisiae* or *E. coli*) was co‐encapsulated with the pathogen (*S. aureus*) and a fluorescent label for dead cells.^[74]^ After incubation, the emulsions were ruptured and the cells were sorted using FACS, allowing the identification of yeast or bacteria with bactericidal properties. In a model sort with a ratio 1:10 000 of positive control yeast (secreting the bacteriolytic enzyme lysostaphin and constitutively expressing yEGFP) and negative control yeast (ineffective against *S. aureus,* non‐fluorescent), the authors reported a complete enrichment over three sorting rounds. Applying a similar methodology, *E. coli* cells encapsulated in hydrogel emulsions were screened for pBAD promoter activity.^[75]^ In this study, a library of single *E. coli* cells expressing GFP were encapsulated in hydrogel emulsions using a microfluidic device. Depending on the promoter sequence, differences in GFP expression allowed FACS sorting based on GFP fluorescence. After sorting and enzymatic digestion of the agarose, the microcolonies were plated on agar plates and analyzed to find an averaged 1.25‐fold improvement in protein expression in one round of screening. In a recent study, Fischlechner et al. described the formation of gel beads with a polyelectrolyte shell. This shell led to the retention of significantly smaller molecules, with a molecular weight cutoff 200‐fold lower than gel beads reported previously. They reported the co‐encapsulation of single *E. coli* cells expressing a variant of a phosphotriesterase and a fluorogenic substrate. Subsequent lysis of the *E. coli* in the gel beads released the active enzyme catalyzing the hydrolysis of a phosphotriester to yield a fluorescent product. The beads retained the fluorescent product and the beads containing the most active variants were selected using FACS at rates >10^7^ Hz. A variant with almost twentyfold higher *k*_cat_/*K*_M_ could be identified in a single round.^[73]^ Similar approaches have been used to evolve production hosts for industrially relevant enzymes: improved overexpression and 1.3‐fold higher secreted amounts of xylanase by *P. pastoris* in gel microdroplets was recently reported by Ma et al.^[76]^

### 2.3 Technology Advances III: On‐Chip Observation, Manipulation, and Sorting of Droplets

**Figure 8:**
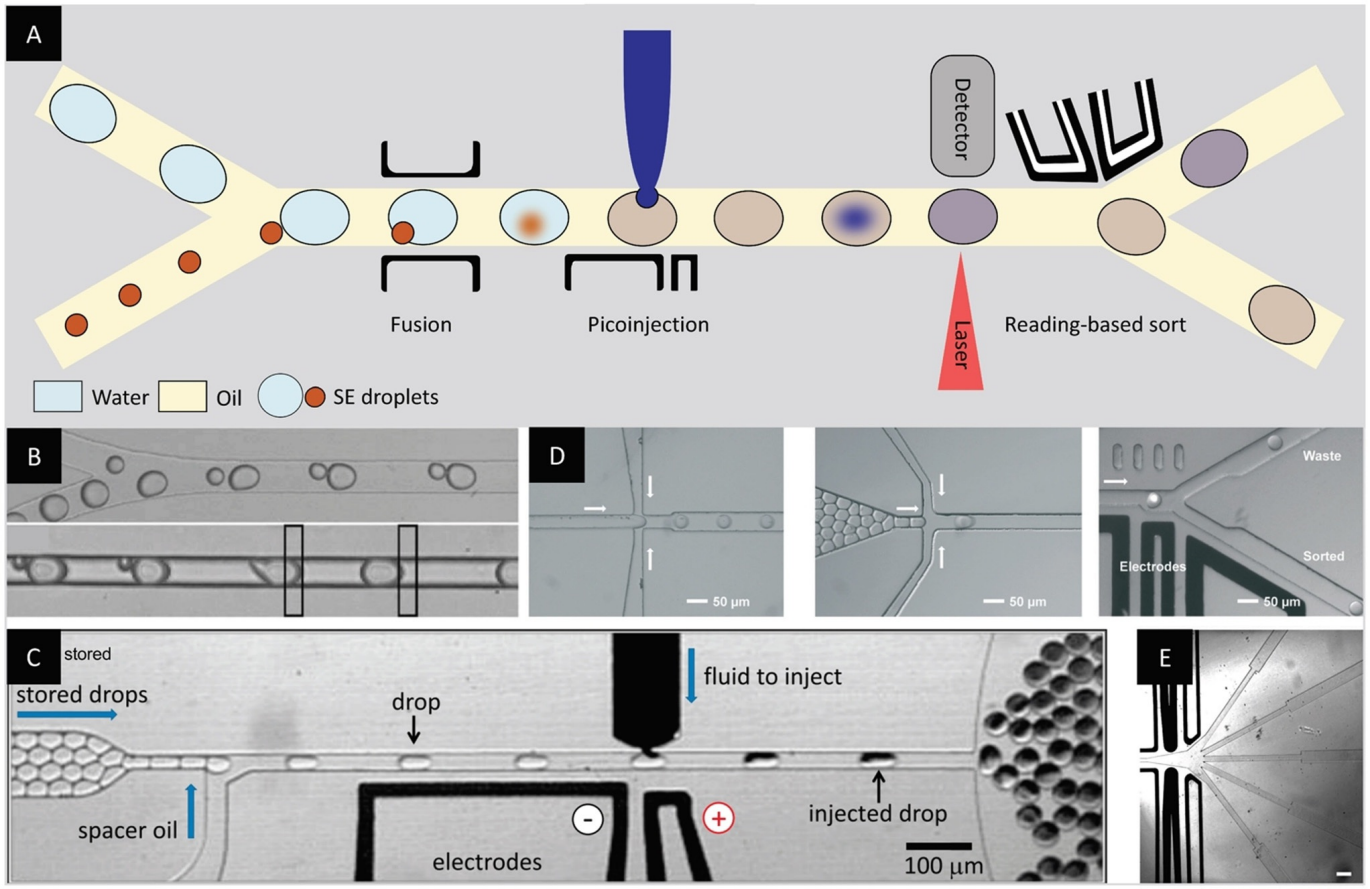
Manipulation, observation and sorting of droplets on‐chip. (A) Integrated electrodes allow the controlled addition of a reagent to pre‐formed droplets at a later time‐point, either by fusing droplets (orange) or by picoinjection (blue). Droplet fluorescence or absorption can be monitored on‐chip and another set of integrated electrodes can sort the droplets in different channels based on the reading. (B) Micrograph of the electrocoalescence of droplet pairs.^[79]^ (C) Micrograph of the addition of reagent to pre‐formed droplets using picoinjection.^[80]^ (D) Micrographs of droplet production on‐chip, followed by the droplet reinjection in a second chip, using oil as spacer, and the droplet sorting by dielectrophoresis based on fluorescence detection.^[81]^ (E) Micrograph of a sorting junction designed for 5‐ways sorting of droplets. Scale bar: 200 μm.^[82]^ (Images reprinted with permission from (B) AIP Publishing LLC, (C) National Academy of Science USA, (D) The Royal Society of Chemistry. Image reprinted (E) from ref. [82]).

Most of the technological developments described so far were optimized for one‐step processes and reactions. However, many reactions require multiple steps where the addition of new reagents is required or the different reaction conditions are not compatible with each other. With the development of droplet‐based microfluidics, more options for generation, fusion, control, and analysis of droplets have emerged (Figure 8 A).[^68^, ^77^]

An important advancement of droplet‐producing microfluidic devices consists in the integration of electrodes generating an electric field across microfluidic channels. In an early study, Chabert et al. investigated the electrocoalescence of w/o pair droplets as a tool for reagent addition. Using AC fields, they succeeded in displacing and merging droplets with a diameter of 600 μm under static and flow conditions.^[78]^ Another study established the high‐throughput electrocoalescence of pair droplets in a PDMS microfluidic chip (Figure 8 B).^[79]^ Two flow streams with droplets of different diameters (13–50 μm) merged into a single channel with downstream electrodes generating an electric field. As the droplet velocity is size‐dependent, the size mismatch allowed the smaller and larger droplets to form pairs upon contact and led to the subsequent electrocoalescence as the pair passed through the electric field. The method was illustrated by determining the *k*_cat_ of an enzymatic reaction through the encapsulation of β‐galactosidase and its fluorogenic substrate, resorufin‐β‐D‐galactopyranoside, in pair droplets. Pioneering studies led to the development of controlled reagent injection to droplets with higher throughput. Abate et al. proposed the use of picoinjectors to add reagents to droplets at frequencies of several thousand Hertz (Figure 8 C).^[80]^ Recent developments for precise reagent delivery inside w/o droplets still involve electric fields and more complex systems such as the rupture of triple emulsions^[83]^ or the use of a three‐phase flow.^[84]^ Yet, the use of electrodes is not restricted to droplet merging but also allows the displacement of droplets.[^37^, ^85^] In an innovative study, Ahn, Kerbage et al. reported on a droplet sorter based on the use of dielectrophoretic forces to direct droplets towards either side of a microfluidic junction.^[86]^

In parallel to the development of droplet manipulation, several groups focused on on‐chip fluorescence detection for reaction monitoring in droplets.^[87]^ In an early study, Dittrich et al. encapsulated ivTT mixture and red‐shifted GFP‐encoding (rsGFP) genes in w/o droplets and monitored the protein expression on‐chip using fluorescence spectroscopy.^[88]^ The combination of sensitive fluorescence detection and dielectrophoretic sorting led to the development of the first fluorescence‐activated droplet sorting (FADS) device.^[36]^ In this joint effort of the Weitz and Griffiths groups, *E. coli* cells expressing either β‐galactosidase or an inactive variant were co‐encapsulated with a fluorogenic substrate. The groups sorted the droplets based on enzymatic activity at a rate of 300 Hz with a low error rate. In a later study by the same authors, a system with three chips for droplet production, reagent addition via pair droplet fusion, and fluorescence intensity based sorting was used for the kinetic monitoring of in vitro translated laccase.^[89]^ Decoupling the processes made it possible to handle droplets at different rates, 7000 Hz for droplet production and 3000 Hz for droplet merging.

A similar decoupled process was used to enrich an active variant of in vitro translated β‐galactosidase.^[90]^ There, the droplets containing active variants were merged on‐chip with an aqueous stream for easier retrieval of the genes. Similarly, Svahn and co‐workers reported on the enrichment of a yeast strain based on its enzyme production using FADS. They achieved an enrichment close to the theoretical maximum and identified a clone with twofold increase in amylase production after a single round of screening (Figure 8 D).^[81]^ In a similar study, Ostafe et al. used FADS to enrich cellulase‐producing yeasts from an inactive cell population with an enrichment factor of up to 300‐fold.^[91]^ Recent improvements of FADS devices involve multiway sorting: Frenzel et al. proposed a chip allowing for droplet sorting in four outlets at a maximal throughput of 2–3 Hz.^[92]^ A later study by Caen et al. described a five‐way sorting system with an almost 100‐fold higher throughput (Figure 8 E).^[82]^ Recently, a microfluidic chip for the sorting of w/o droplets based on fluorescence lifetime was reported by Hasan et al.^[93]^ Most of the examples described above for dielectrophoresis‐based sorting rely on the use of fluorogenic substrates. However, recent studies propose label‐free techniques for on‐chip sorting. Alternatives such as interfacial tension based sorting to distinguish between live and dead cells^[94]^ or intelligent image‐based sorting capable of analyzing images and taking sorting decisions in real‐time[^95^, ^96^] offer interesting prospects.

#### 2.3.1 Applications I: In Vitro

An initial effort involving a multistep process was highlighted using the example of a CotA sporulation protein, a laccase from *Bacillus subtilis* catalyzing the oxidation of various aromatic compounds using molecular oxygen as oxidant. Since the laccase assay was incompatible with the in vitro protein expression system, sequential addition of reagents at different time points was required.^[89]^

Following this strategy, Fallah‐Araghi et al. developed a completely in vitro platform for the screening of active *lacZ* genes encoding the enzyme β‐galactosidase starting from single genes. In their study, genes were encapsulated before on‐chip electro‐coalescence and sorting. In a model sort of active *lacZ* genes vs. inactive *lacZmut* with a ratio of 1:100, they reached a 502‐fold enrichment in a single round of screening.^[90]^ Building on these examples, Goto et al. reported a device for the encapsulation and sorting of nanoliter droplets and applied the method on the model screening of an isocitrate dehydrogenase (IDH) from *Streptococcus mutans*. Starting from a library of 10^3^ variants and two rounds of screening, they isolated a variant with about threefold higher activity than wild type.^[97]^

#### 2.3.2 Applications II: In Vivo

Technological progress led to up to 1000‐fold faster screening and a million‐fold decrease in reagent costs as exemplified by the joint efforts of Abate, Baret, Griffiths, and Weitz. In their seminal study, they applied on‐chip droplet generation and dielectrophoretic sorting for the high‐throughput screening of HRP displayed on the surface of yeast cells. After sorting, the droplets were ruptured, thus making the most active yeast cells readily available for the next round of mutagenesis and sorting. With this screening platform, they screened libraries with up to 10^7^ variants and achieved an overall ≈sevenfold improvement in catalytic efficiency over nine rounds. The highest catalytic efficiency reached was ca. 2.5×10^7^ M^−1^ s^−1^, thus approaching diffusion‐limited efficiency (i.e. 10^8^ M^−1^ s^−1^).^[98]^ Similar setups involving yeast encapsulated in single emulsions were used, for example, for the evolution of thermostable xylanase with improved activities (up to 4.7‐fold)^[99]^ or the improvement of yeast cells as production hosts (twofold increase in α‐amylase production).^[81]^

One of the first examples expanding the repertoire to *E. coli* was reported in 2015 by Abate and co‐workers. They mapped protein sequence–function relationships by combining microfluidics with next‐generation sequencing, and analyzing both sorted and unsorted populations. Starting from a library of 6×10^7^ variants, they enhanced glycosidase activity at higher temperatures in a single round of mutagenesis. The deep mutational scanning revealed regions which might be crucial for glycosidase activity, but also highlighted known patterns with mutational tolerance which were in accordance with examples from the enzyme family.^[100]^

**Figure 9:**
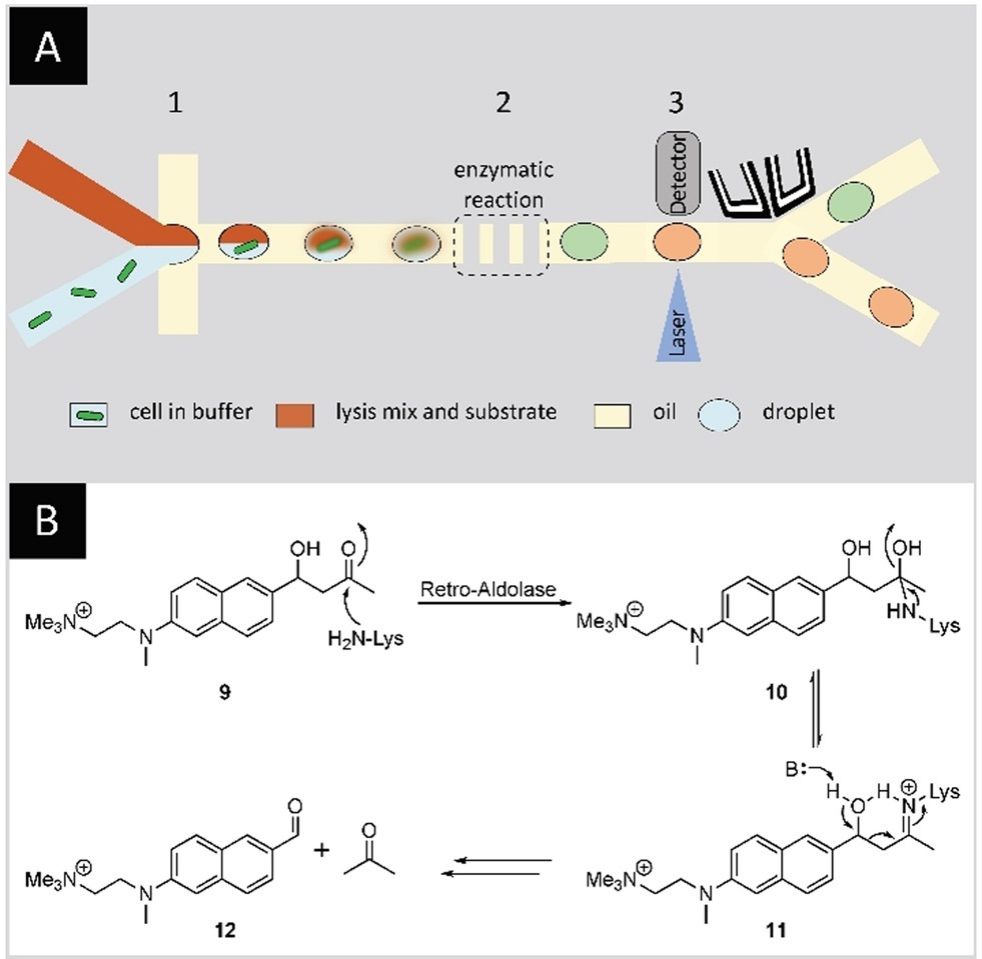
Directed evolution of a retro‐aldolase using microfluidics‐based FADS. A) Microfluidics‐based FADS. *E. coli* are encapsulated with a substrate/lysis mix in w/o droplets (1). The cells are lysed in the droplets making the expressed retro‐aldolase (orange) readily available and converting the aldol substrate (red) into a fluorescent product (green) (2). Finally, the droplets are sorted on‐chip by activating the sorting electrodes when the fluorescence signal exceeds a certain threshold (3). B) Retro‐aldolase‐catalyzed cleavage of a charged methodol derivative (**9**) via an enzyme‐bound Schiff base intermediate (**11**) yields a fluorescent naphthaldehyde derivative (**12**) and acetone. The positive charge on the substrate/product ensures their retention in the droplets.^[38]^

Directed evolution is especially versatile if the initial catalytic activities are low, as for example for de novo designed biocatalysts. In another study, Hilvert and co‐workers applied a microfluidics‐based screening coupled to FADS to evolve a retro‐aldolase by amine catalysis.^[101]^ A previously designed retro‐aldolase capable of cleaving a carbon–carbon bond in a non‐natural substrate ((±)‐methodol) via an enzyme‐bound Schiff base intermediate (**11**) showed modest catalytic efficiency (*k*_cat_/*K*_M_=0.19 M^−1^ s^−1^) and enantioselectivity (*ee*=33 % for (*S*)‐methodol).^[13]^ It was used as the starting point for the directed evolution campaign and the authors were able to significantly improve the catalytic activity. *E. coli* cells expressing the protein of interest in the cytoplasm were encapsulated in w/o emulsions with lysis buffer to release the enzyme in the droplet and a fluorogenic, charged methodol derivative (**9**) (Figure 9). Six focused libraries with up to five simultaneously mutated residues were screened and a variant with almost 80‐fold increase in *k*_cat_ was identified in a single round of screening. Strikingly, the same mutations were identified in an earlier study on the same enzyme. This previous study, relying on a medium‐throughput screening campaign using MTPs, required five rounds of directed evolution to install these mutations. The best performing mutant of the microfluidics‐based study was identified after only two rounds of evolution. It included ten mutations and exhibited a 73‐fold increase in *k*_cat_/*K*_M_ and tenfold preference for (*S*)‐methodol.^[101]^ The kinetics of the catalytic system were further optimized to give >10^9^ rate enhancement, thus approaching Class I aldolase activities (natural enzymes catalyzing reversible carbon–carbon bond‐forming reactions) and accommodating a wider substrate scope.^[102]^ The same group reported the isolation of an active cyclohexylamine oxidase (CHAO) identified from a single screening round of a library with 10^7^ variants. They remodeled the active site of CHAO, achieving up to 960‐fold increase in catalytic efficiency thus approaching the wild type levels of activity for a non‐natural substrate.^[103]^

## 3 Double Emulsions

Water‐in‐oil‐in‐water (w/o/w) double emulsions can overcome some of the challenges remaining with single emulsions, such as limited stability (e.g., shrinkage of droplets) and the need for rapid on‐chip analytical methods. Although their creation requires an additional emulsification step,^[104]^ the handling of w/o/w double emulsions on‐ and off‐chip offers intriguing advantages; in particular, the compatibility with commercially available FACS sorting devices is noteworthy.[^105^, ^106^]

### 3.1 Technology Advances I: Bulk Emulsification and Development of Compatible Assays

**Figure 10:**
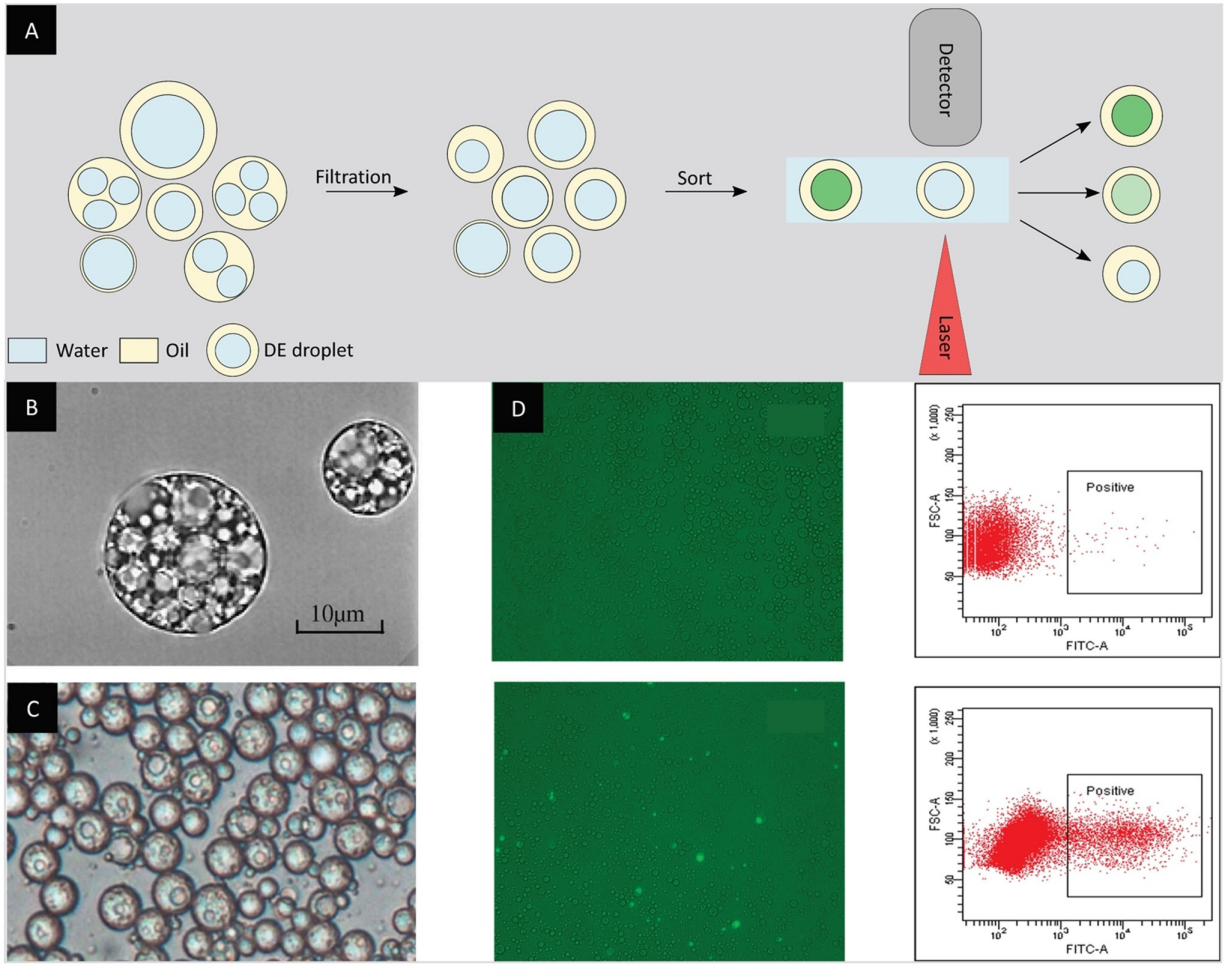
Bulk emulsification for the formation of double‐emulsion droplets. A) Double‐emulsion droplets formed by bulk emulsification are polydisperse and can contain multiple inner aqueous phase compartments. An additional filtration step can improve the sample homogeneity and lead to more reliable FACS sorting. B) Micrograph of double‐emulsion droplets resulting from bulk emulsification.^[109]^ C) Micrograph of double‐emulsion droplets after membrane extrusion.^[112]^ D) Micrograph of double emulsions encapsulating either *E. coli* cells containing a plasmid for the expression of esterase on the cell surface or negative *E. coli* cells. The use of a fluorescein‐based fluorogenic substrate allows the identification and sorting of double emulsions with cells displaying esterase activity as shown in the FACS plot.^[112]^ (Image reprinted with permission from (B) Elsevier. Images reprinted from (C) and (D) ref. [112]).

Much like for single emulsions, the first methods developed for the formation of double emulsions were based on bulk emulsification using stirring or emulsifiers.^[107]^ Double emulsions formed using these strategies are polydisperse and the inner aqueous phase compartment usually consists of multiple compartments (Figure 10 B). In an early study, Ficheux et al. scrutinized the stability vs. coalescence of the inner aqueous compartments of double emulsions. Their research highlighted that the double emulsion's stability is mostly affected by the surfactant type (water‐ or oil‐soluble) and concentration, and can vary on a timescale that ranges from minutes to months.^[31]^ Following these findings, other research groups investigated the stability of double emulsions with different surfactants and composition of outer aqueous phases.[^108^, ^109^] A two‐step emulsification process using a Couette mixer for the formation of quasi‐monodisperse double emulsions with multiple aqueous compartments was proposed by Goubault and co‐workers.^[110]^

More recent studies involving double emulsions generated in bulk focus on improving the compatibility of double emulsions with different screening methods. In their study, Prodanovic et al. proposed a fluorescent cascade assay in double emulsions for sorting enzyme libraries by FACS. The assay allowed the screening of a glucose oxidase gene library with 10^4^ mutants based on the hydrogen peroxide production with a 50–200‐fold enrichment factor.^[111]^ To overcome the limitations imposed by the polydispersity of bulk double emulsions, Ma et al. improved the production method by using membrane extrusion, leading to the generation of more uniform double emulsions. The advantages of this method were illustrated by enriching a population of *E. coli* cells with esterase activity more than 300‐fold (Figure 10 C and Figure 10 D). The method was further applied to the directed evolution of a thermophilic esterase AFEST, resulting in a twofold improvement in catalytic activity as well as the identification of several mutants with *k*_cat_/*K*_m_ values approaching diffusion‐limited efficiency.^[112]^

#### 3.1.1 Applications: In Vitro and In Vivo

**Figure 11:**
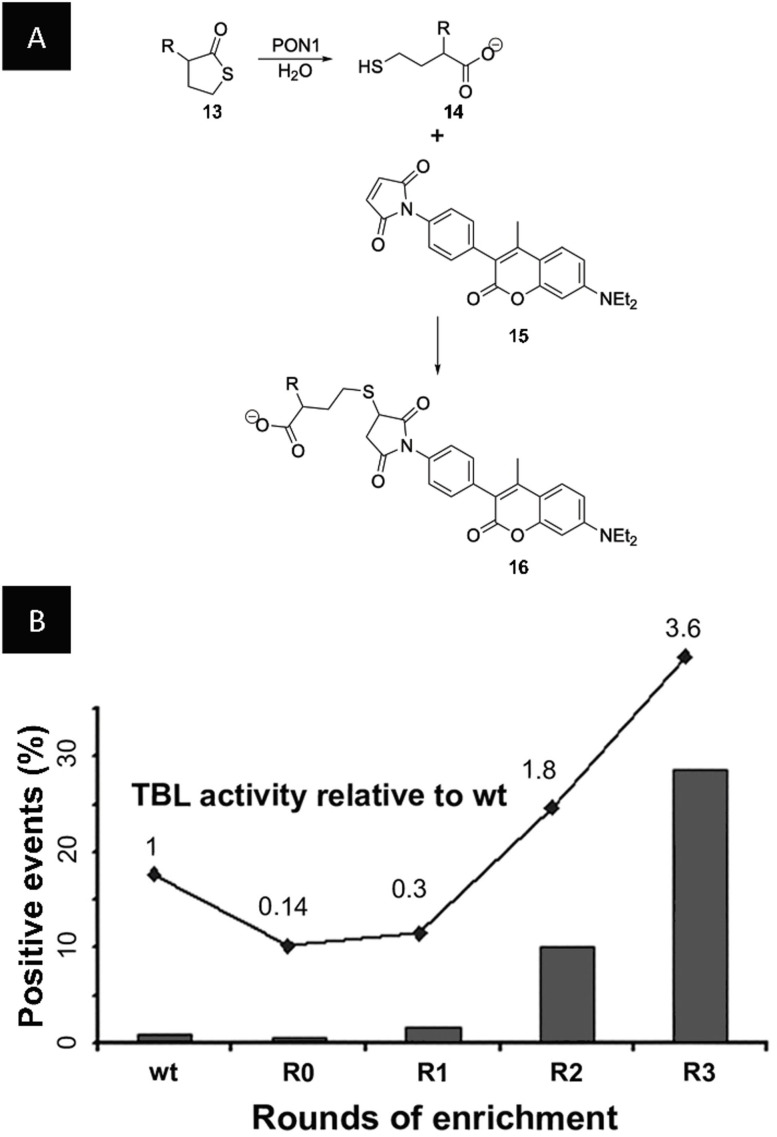
A) PON1 hydrolyzes γTBL (**13**) to the corresponding thiol **14**. *N*‐(4‐(7‐diethylamino‐4‐methylcoumarin‐3‐yl)phenyl)maleimide (CPM, **15**) reacts with the free thiol to form a fluorescent product (**16**). B) Development of the fluorescence intensity over three rounds of enrichment. TBLase activity was determined in the crude lysate of the selected pool and normalized to the activity of wild type PON1 (wt).^[32]^ (Figure reprinted with permission from (B) Elsevier.).

The first application of double emulsions to directed evolution involved the model enrichment of the above‐mentioned FolA/M.HaeIII system. Positive w/o/w droplets containing FolA and the fluorescence marker FITC‐BSA and negative w/o/w droplets containing M.HaeIII and BSA were produced separately and mixed in different ratios before sorting with a commercial FACS device. The positive and negative droplets were mixed in a 1:100 ratio and within one round of sorting a ≈30‐fold enrichment was observed.^[113]^ Using the same technique, Griffiths and co‐workers reported the first completely in vitro directed evolution campaign using double emulsions for the evolution of Ebg, a protein of unknown function. Starting with negligible activity, they screened a library of 2×10^6^ members over four rounds of directed evolution and identified variants with β‐galactosidase activity with at least 300‐fold higher *k*_cat_/*K*_M_ values compared to wild type Ebg.^[114]^ The first study involving in vivo directed evolution in double emulsions was based on *E. coli* surface‐displayed serum paraoxonase 1 (PON1). PON1 is a mammalian enzyme capable of hydrolyzing a broad range of substrates, in particular the homocysteine thiolactone, and thereby eliminating toxic metabolites. In a two‐step process using a homogenizer, single emulsions containing *E. coli* with surface‐displayed PON1 were produced. The substrate (**13**) and a thiol‐detecting dye (**15**) were then added via the oil phase, and a subsequent emulsification step led to the generation of double emulsions. PON1 was evolved for the hydrolysis of thiobutyrlactones (TBLs, **13**), a generally poor substrate of PON1 (*k*_cat_/*K*_M_=75 M^−1^ s^−1^) (Figure 11 A). Starting from a library of 10^6^ mutants, three cycles of screening led to a variant with up to one hundredfold higher TBLase activity (*k*_cat_/*K*_M_=10^4^ M^−1^ s^−1^) (Figure 11 B).^[32]^ A variant of the same enzyme, rePON1, was further investigated as a target against nerve agents based on organophosphates. By applying random and targeted mutagenesis, coupled to high‐throughput FACS screening and MTP assays, mutants capable of hydrolyzing cyclosarin with *k*_cat_/*K*_M_ ≈10^7^ M^−1^ s^−1^ were identified. These findings were also applied to prophylactic studies involving mice, where the identified hits exhibited considerable protection against a lethal dose of a cyclosarin derivative.^[115]^ Further applications include the directed evolution of 1) β‐glucosidase leading to a twofold increase in lactose specificity and catalytic turnover rates,^[116]^ 2) the development of a model protease with 1.6‐fold increased resistance towards the inhibitor antipain dihydrochloride,^[117]^ and 3) the screening of a cellulase mutant library with the identification of variants with over 13‐fold increased specific activity compared to wild type.^[118]^

**Scheme 2:**
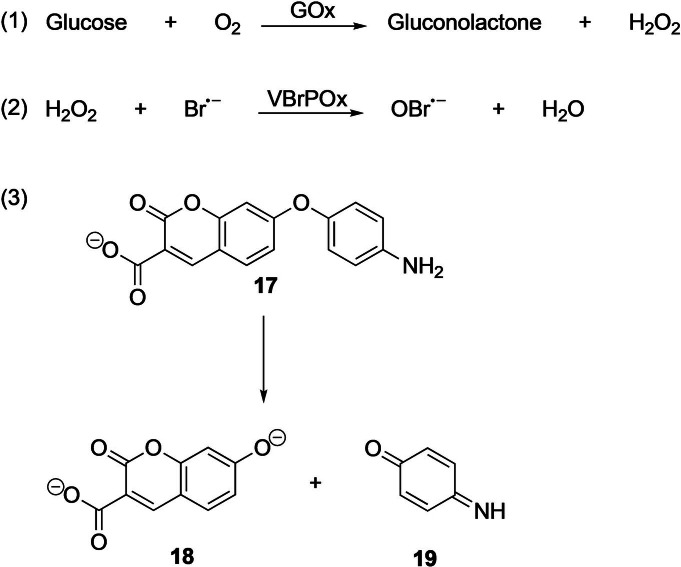
A vanadium bromoperoxidase coupled (ViPer) fluorescent assay for the directed evolution of GOx and subsequent sorting.^[111]^

Prodanovic et al. highlighted the versatility of this screening platform by expanding this technology to in vivo encapsulated yeast in combination with a vanadium bromoperoxidase coupled fluorescence assay (ViPer) to detect H_2_O_2_ (Scheme 2). In the assay, H_2_O_2_ was used by the bromoperoxidase to produce hypobromide, which reacted with a fluorogenic probe to release fluorescent coumarin. Using this approach, a 200‐fold enrichment of active GOx was identified in a single screening round starting from a library of ≈10^4^ variants.^[111]^ Similarly, cellulase activity was evolved to achieve a 12‐fold enrichment of the active variant in a single round.^[119]^

### 3.2 Technology Advances II: On‐Chip Formation and Stability Optimization for High‐Throughput Sorting

**Figure 12:**
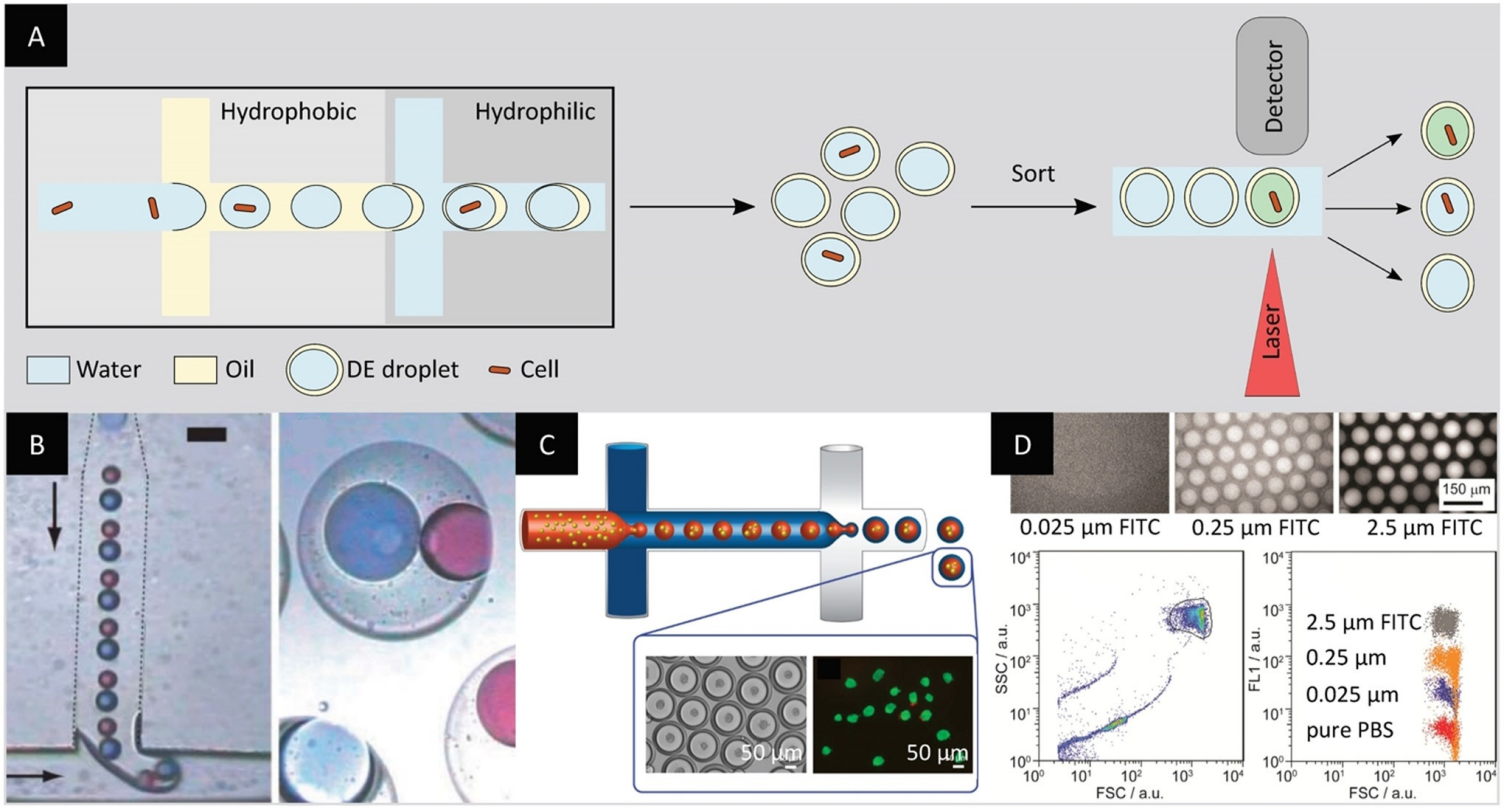
Microfluidics‐based formation of double‐emulsion droplets. A) Double‐emulsion (DE) droplets are formed in a microfluidic chip using two consecutive flow‐focusing junctions. The first emulsification step requires hydrophobic channel walls, while the second step requires hydrophilic channels. The DE droplets can be sorted by conventional FACS. B) Micrograph of the formation of DE droplets with two aqueous compartments. Scale bar: 100 μm.^[122]^ C) DE formation and encapsulation of cells for the growth of multicellular spheroids.^[129]^ D) Micrographs of DEs containing discrete concentrations of fluorescent dye, and FACS plot displaying the discrimination between the different DE populations.^[132]^ (Images reprinted with permission from (B) the Royal Society of Chemistry, (C) Springer Nature Limited. Image adapted (D) from ref. [132]).

Producing double emulsions on‐chip greatly improved the monodispersity and the control over the number of inner aqueous phase compartments.^[120]^ Furthermore, it greatly improved the droplet sorting efficiency and throughput. However, the production of double‐emulsion droplets on‐chip is more challenging than the formation of single emulsions as it requires different surface‐wetting properties for each emulsification step. The microfluidic junctions need to be hydrophobic for the first w/o emulsion and hydrophilic for the second w/o/w emulsion. Various strategies have been investigated to address this challenge, such as decoupling the two emulsification steps, using coating solutions, or building the chip from different materials. In a pioneer study, Okushima and co‐workers proposed several design options allowing for either 1) decoupled emulsification steps in two quartz and Pyrex glass chips or 2) double emulsification on one single Pyrex glass chip. For both designs, the double emulsions were produced using T‐junctions. The requirements for different channel surface properties at the junctions were satisfied by coating the first junction hydrophobically with a silane‐coupling agent. Using these devices, the authors produced monodisperse double emulsions of about 100 μm in diameter at a rate of 22 Hz (Figure 12 B).[^121^, ^122^] Another study reported the formation of double emulsions at higher throughput using glass microcapillaries. Droplets 10–50 μm in diameter were produced at rates ranging from 100–5000 Hz. The authors further highlighted the potential of their device by controlling the size of the inner water droplet and oil shell of the double emulsion.^[123]^

The need for rapid prototyping and simple microfabrication led the field towards the use of PDMS‐based microfluidic devices. In a first study on PDMS surface modification using plasma polymerization, the authors achieved selective hydrophilic coating and subsequent formation of double emulsions with a T‐junction.^[39]^ The formation of double emulsions with controlled oil shell thickness was reported by Abate et al.^[40]^ A PDMS chip with two consecutive flow‐focusing junctions was selectively coated using a flow‐confinement technique.^[124]^ Similar devices with a step structure at the second flow‐focusing junction were developed to facilitate the second emulsification.[^125^, ^126^] Due to the critical nature of the coating and the precision required for the wettability patterning, different strategies for coating or decoupling the two emulsification steps were developed and are described in detail elsewhere.[^127^, ^128^]

Double emulsions formed on‐chip initially found applications in cell culturing and in vitro protein expressions (Figure 12 C).[^47^, ^129^, ^130^] Notably, Zhang and co‐workers encapsulated *E. coli* in monodisperse double emulsions produced on two decoupled PDMS chips. They studied bacterial growth and protein expression by addition of the inducer in the outer aqueous phase and utilizing diffusion across the oil layer.^[131]^

The ability to use FACS on double emulsions constituted an essential advance for improved compatibility of double emulsions in the context of directed‐evolution studies (Figure 12 D). Efforts were therefore invested in studying the deformation of double emulsions in FACS devices and in identifying suitable surfactants to ensure droplet stability.[^132^, ^133^]

#### 3.2.1 Applications: In Vitro and In Vivo

These technological advances led to the development of a platform for single‐cell and enzymatic activity‐screening. Terekhov and Smirnov et al. combined FACS sorting of double emulsions with downstream next‐generation sequencing and liquid chromatography–mass spectrometry (LC‐MS) analysis of secretome and proteome. In their comprehensive study, the authors used a two‐step on‐chip emulsification process to perform enzyme screenings with different organisms. They succeeded in sorting active yeast cells displaying an enzyme on their membrane from a non‐active population using a fluorogenic substrate. Several mixing ratios—up to 1:10^5^—were investigated, and the authors achieved maximum enrichments for the low dilutions and significant enrichment for the highest dilution. They further illustrated the potential of their platform for distinguishing between different enzymatic activities and between different levels of enzymatic activity. Finally, the cell‐to‐cell interaction between different organisms was investigated using yeast and bacterial cells.^[134]^

First studies highlighting the power of double emulsions include the efficient enrichment of active wild‐type arylsulfatase from a low‐activity mutant. Enrichment factors of 800‐ and 2500‐fold, starting from populations of 0.1 % and 0.01 % active cells, respectively, have been reported.^[135]^ Using a fluorescent reporter system which gave a positive signal upon full‐length amplification of the template DNA by the target polymerase, Larsen et al. expanded polymerase function to non‐natural genetic polymers. After establishing the approach by enriching a model engineered polymerase ≈1200‐fold, the screening method was applied to evolve a manganese‐independent α‐l‐threofuranosyl nucleic acid (TNA) polymerase. In merely one round of selection, they identified a manganese‐independent TNA polymerase with higher fidelity and ≈14‐fold improved activity.^[41]^

## 4 Latest Developments and Label‐Free Methods

**Figure 13:**
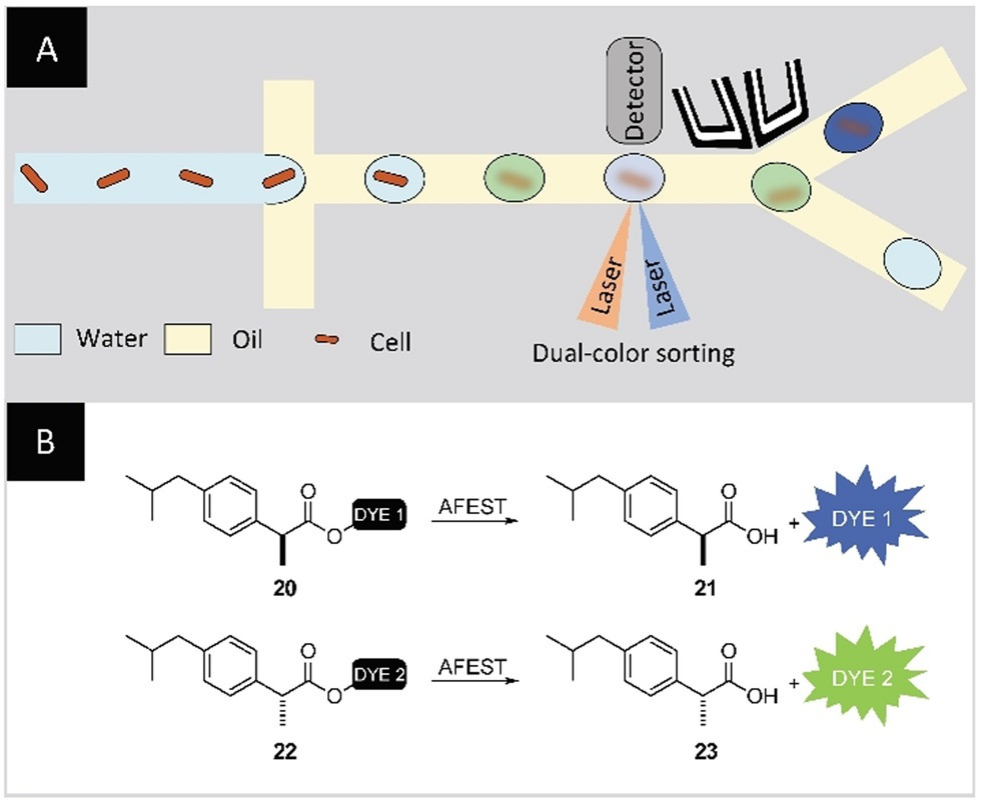
A) Schematic representation of the dual‐channel microfluidic droplet screening (DMDS). B) (*S*)‐Ibuprofen and (*R*)‐ibuprofen modified with two different fluorophores, and the enzymatic reaction yielding two different fluorescent signals. Substrate **20** is used as the selection substrate and substrate **22** as the counter‐selection substrate. To improve the enantioselectivity of AFEST towards (*S*)‐ibuprofen, variants with increased fluorescence signal for dye 1 but lower fluorescence signal for dye 2 were selected using on‐chip sorting.^[142]^ (Image adapted from ref. [142]).

Recent progress on the microfluidic/technology side focus on optimizing existing tools and methods, aiming at a more straightforward use and more reproducible results. Notably, Sukovitch, Kim, and co‐authors proposed a method to simplify double‐emulsion production while conserving the monodispersity. They coupled single‐emulsion production on‐chip with a second bulk emulsification step, circumventing the complex coating process required for double‐emulsion chips.^[136]^ New ways of delivering reagents inside single or double emulsions, mainly by adapting the surfactant type and concentration, have been characterized by several research groups.[^137^, ^138^, ^139^, ^140^, ^141^] In parallel, substantial advances have been achieved in expanding the screening capabilities of microfluidic platforms. In a groundbreaking study, Ma and co‐workers reported a dual‐channel microfluidic droplet screening system (DMDS). This system enables the simultaneous sorting of w/o droplets according to two properties of a target enzyme using two different fluorogenic substrates (Figure 13 A). The efficiency of the platform for the screening of complex enzymatic properties was illustrated with the directed evolution of a highly enantioselective esterase from *Archeoglobus fulgidus* (AFEST) (Figure 13 B).

After five rounds of evolution, a variant with 700‐fold improvement in enantioselectivity for (*S*)‐profens was obtained.^[142]^ In a recent study, Brower et al. introduced a comprehensive FACS‐based method to sort and isolate double‐emulsion droplets produced on‐chip. Their method allows for the encapsulation of a variety of mammalian cells and sorting at throughputs >10 kHz while maintaining the w/o/w droplets’ integrity, followed by retrieval of genetic material.[^143^, ^144^]

Although fluorescence detection is still the gold standard for assaying enzymatic activities in droplets, reports investigating other techniques have recently gained attention. These techniques give access to enzyme characteristics without requiring the use of a fluorogenic substrate. A first example reported by Gielen et al. introduced a microfluidic device for absorbance‐activated droplet sorting (AADS). With this device, the authors evolved a phenylalanine dehydrogenase over two rounds of screening and found a variant with 4.5‐fold increased activity and >10 °C increased thermostability.^[145]^ Similarly, passive sorting strategies, such as sorting by interfacial tension, will allow novel types of assays, where changes in droplet content translate into different droplet properties.^[94]^

**Figure 14:**
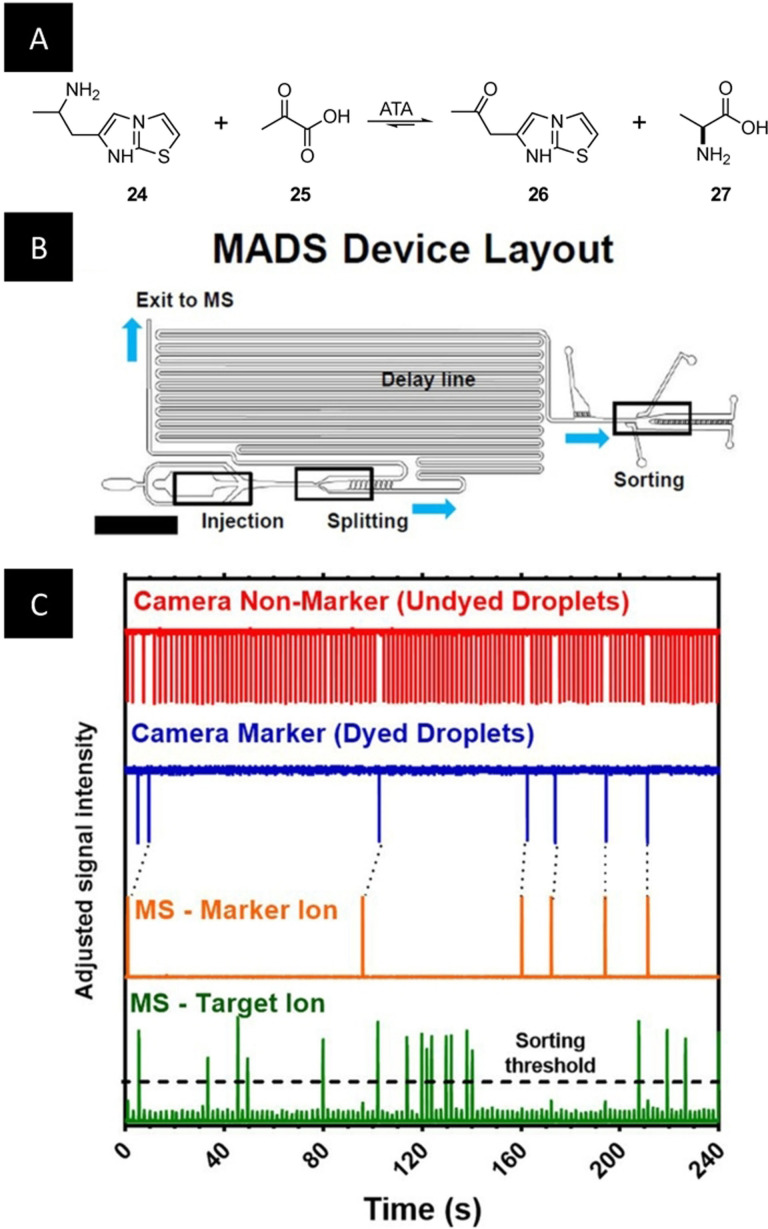
A) The transaminase activity of ATA 117 is screened by evaluating the transformation of the non‐native ATA substrate (**24**) and pyruvate (**25**) to the ATA product (**26**) and alanine (**27**) after ivTT. B) Schematic representation of the mass‐activated droplet sorting (MADS) device. Nanoliter‐sized droplets are injected in the bottom left region (“injection”) and are split asymmetrically (“splitting”). While the larger droplet travels directly to the mass spectrometer, the smaller droplet flows through the delay line. The smaller droplet is sorted using a dielectrophoretic sorter (“sorting”) according to the sorting decision made using the MS‐signal. C) For the MADS device to function, three different samples are analyzed in parallel. Inactive and therefore uncolored droplets are recognized by a camera by pattern tracing (red). Marker droplets for synchronization contain food color and are detected by the camera (blue). The signals are synchronized with the MS signal of the marker‐ion (orange). After synchronization, the MS signal of the target ion (green) is used to make a sorting decision.^[151]^ (Image reprinted with permission from Wiley‐WCH Verlag GmbH & Co KGaA.).

One of the most promising and widely applicable alternatives to fluorescence‐based readouts is mass spectrometry (MS), which allows label‐free multiplexed characterization of several analytes. In the past years, several groups have illustrated the compatibility of droplet microfluidics with electrospray ionization (ESI)‐MS[^146^, ^147^] or matrix‐assisted laser desorption/ionization (MALDI)‐MS for the analysis of enzyme secretion of yeast cells.^[148]^ Notably, the Kennedy group has reported the coupling of w/o droplets with high‐throughput MS for the in vitro screening of enzyme inhibitors and activators.[^149^, ^150^] With their methods, droplets can be directly injected in the ESI‐MS at a throughput of almost 1 Hz. In a follow‐up study, the authors increased their platform throughput more than threefold. The method was applied to the screening of transaminase libraries and further highlighted the compatibility of their system with in vitro translation–transcription of proteins (Figure 14 A).

The most recent advance in this field concerns the screening of enzymatic reactions with an innovative method, termed MADS (mass‐activated droplet sorting). MADS combines MS analysis with FADS and benefits both from the high sensitivity of MS and from the possibility of collecting the sample allowed by FADS. The MADS device allows for droplet production and splitting of each droplet in two. One fraction is analyzed by ESI‐MS while the second fraction follows a delay channel leading to a FADS electrode (Figure 14 B). The ESI‐MS results allow the active sorting of the second fraction with a throughput of 0.7 Hz (Figure 14 C). The authors applied their methods to the activity‐based screening of a model transaminase library expressed in vitro.^[151]^

## 5 Outlook

Moving away from model enrichments and display of platform capabilities, many research groups are now working on improving enzymes with industrial or medical relevance.[^65^, ^74^, ^76^, ^150^, ^152^, ^153^] Besides the evolution of natural enzymes, the toolkit of directed evolution is expanding to artificial enzymes to introduce non‐natural reactivities urgently needed in the pharmaceutical industry,^[152]^ de novo designed enzymes to understand and reengineer enzyme active sites, and, more recently, machine‐learning‐assisted directed evolution.^[154]^ Furthermore, as fluorogenic substrates cannot always be synthesized, novel strategies for fast, but non‐fluorescence‐based detection will be critical for future developments.

The advances in droplet microfluidics over the past 20 years have permitted decisive steps towards the discovery of enzymes with new or improved functionalities. The higher throughput and facilitated sorting enabled the directed evolution of libraries of increasing sizes at a significantly reduced time and material consumption. Droplet microfluidics paved the way to automated workflows and faster screenings, and enabled the use of conventional FACS, accessible in most biology institutes. Close collaborations between engineering groups and chemistry or biology research groups showed a synergetic effect by allowing successful large campaigns. To continue on this prosperous avenue, microfluidics systems must be further simplified to enable robust operation of microfluidic devices by non‐experts. Cheap, commercially available microchips will further lower the hurdles to exchange standard tools for microfluidic systems.^[155]^

With more and more groups working on directed evolution using microfluidic systems, the spectrum of applications and assays will broaden. We believe that the joint effort from these two fields holds great promise, and we are looking forward to the new innovative developments that will emerge from collaborations between engineers and biochemists in the future!

## Conflict of interest

The authors declare no conflict of interest.

## Biographical Information

*Ariane Stucki holds a Master's degree in Engineering Physics and a Minor in Biomedical Technologies from Ecole Polytechnique Fédérale de Lausanne (EPFL). She is currently a PhD student in the Department of Biosystem Science and Engineering, ETH Zürich, in the Bioanalytics Group under the supervision of Prof. Dr. Petra Dittrich. Her projects focus on the development of droplet‐based microfluidic tools for applications in chemistry and biology*.

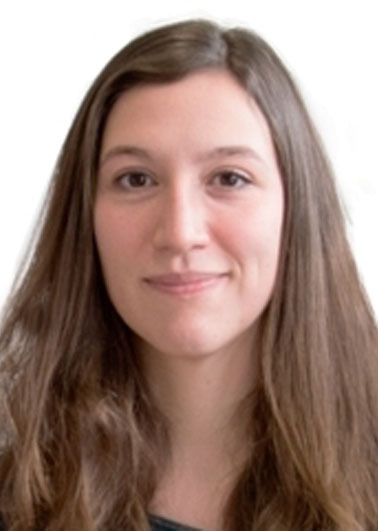

## Biographical Information

*Thomas Ward obtained his PhD at ETH Zürich. Following a decade dedicated to homogeneous catalysis, he switched his focus to the development of artificial metalloenzymes. He is Professor at the University of Basel and Director of the NCCR Molecular Systems Engineering. In this context, he has been collaborating with engineering colleagues to develop a microfluidics platform to evolve artificial metalloenzymes*.

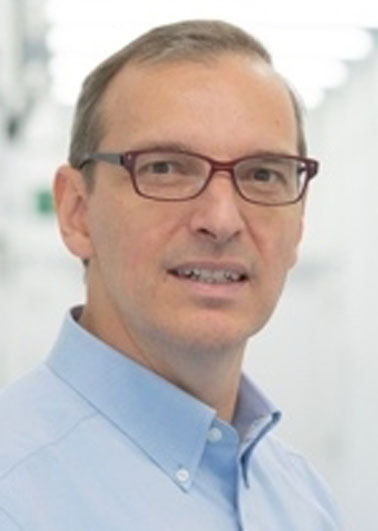

## Biographical Information

*Jaicy Vallapurackal completed her Bachelor and Master studies at the Department of Chemistry, University of Basel and is currently a PhD student in the Department of Chemistry under the supervision of Prof. Dr. Thomas R. Ward. Her project is focused on the high‐throughput directed evolution of artificial metalloenzymes and in vivo screening of E.  coli using a microfluidics‐based screening assay*.

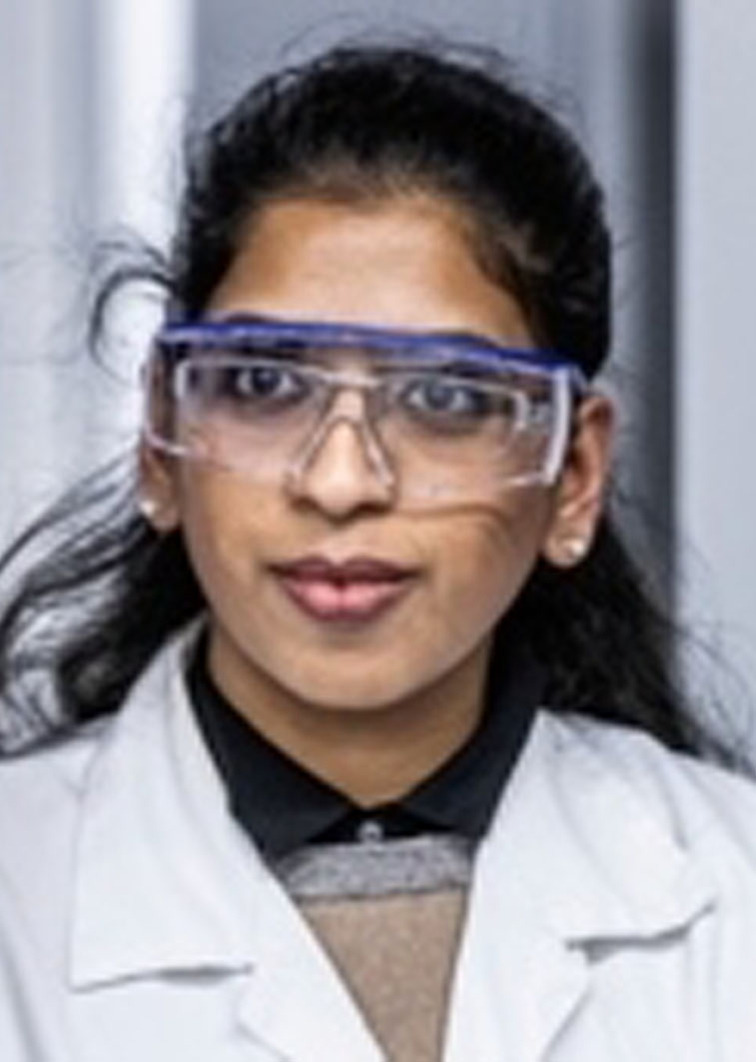

## Biographical Information

*Petra Dittrich is Associate Professor for Bioanalytics at the Department Biosystems Science and Engineering of ETH Zürich (Switzerland). She obtained her PhD at the Max‐Planck Institute for Biophysical Chemistry in Göttingen (Germany). After a postdoctoral appointment at the Institute for Analytical Sciences (Dortmund, Germany), she became Assistant Professor at ETH Zürich. She develops microfluidic methods for bioanalytical applications and diagnostics*.